# The host transcriptome change involved in the inhibitory effect of exogenous interferon-γ on Getah virus replication

**DOI:** 10.3389/fmicb.2023.1214281

**Published:** 2023-06-28

**Authors:** Jialei Li, Xintao Gao, Xingjian Liu, Tong Wu, Haozhi Song, Weisong Gao, Hong Jia, Yinü Li, Zhifang Zhang

**Affiliations:** ^1^Biotechnology Research Institute, Chinese Academy of Agricultural Sciences, Beijing, China; ^2^Institute of Animal Sciences, Chinese Academy of Agricultural Sciences, Beijing, China

**Keywords:** Getah virus, immune escape, IFN-γ, inhibition of replication, transcriptome change

## Abstract

**Introduction:**

Getah virus (GETV) has become a growing potential threat to the global livestock industry and public health. However, little is known about the viral pathogenesis and immune escape mechanisms, leading to ineffective control measures.

**Methods:**

In this study, the antiviral activity of exogenous interferons (IFNs) was assessed by using western blotting (WB), real-time quantitative PCR (RT-qPCR) and indirect immunofluorescence assay (IFA). The comparative transcriptomics among mock- and GETV-infected (MOI = 0.1) ST cells with or without IFN-γ was performed by RNA-seq, and then the transcriptome profiling of GETV-infected ST cells and key pathways and putative factors involved in inhibitory effect of IFN-γ on GETV replication were analyzed by bioinformatics methods and RT-qPCR.

**Results:**

The results showed that treatment with IFN-γ could suppress GETV replication, and the inhibitory effect lasted for at least 48 h, while the exogenous IFN-α/ω and IFN-λ3 treatments failed to inhibit the viral infection and early replication *in vitro*. Furthermore, the blueprint of virus-host interaction was plotted by RNA-seq and RT-qPCR, showing systemic activation of inflammatory, apoptotic, and antiviral pathways in response to GETV infection, indicating viral hijacking and inhibition of innate host immunity such as IFN-I/III responses. Last and most importantly, activation of the JAK-STAT signaling pathway and complement and coagulation cascades may be a primary driver for IFN-γ-mediated inhibition of GETV replication.

**Discussion:**

These findings revealed that GETV possessed the capability of viral immune escape and indicated that IFN-γ aided in the prevention and control of GETV, implying the potential molecular mechanism of suppression of GETV by IFN-γ, all of which warrant emphasis or further clarification.

## 1. Introduction

GETV, belonging to the genus *Alphavirus* and family *Togaviridae*, is a neglected mosquito-borne arbovirus (Wang et al., 2022). As a causative epizootic agent, GETV can cause moderate illness in horses (Nemoto et al., 2015; Lu et al., 2019), lethal disease in foxes (Shi et al., 2019), reproductive disorders, and fetal death in pigs (Yang et al., 2018). Moreover, serologic surveys and virus isolation showed that the infection might occur in multiple other vertebrate species, including cattle, monkeys, goats, rabbits, kangaroos, poultry, wild birds, and human beings (Marchette et al., 1980; Li et al., 1992).

Owing to the wide range of hosts, multiple routes of transmission, and large-scale business of animal husbandry in China, combined with the analogy analysis of other mosquito-borne viruses such as Zika and dengue virus, the potential threats to animals and public health posed by GETV appear certain, especially in China (Li et al., 2019, 2022; Lu et al., 2020). Furthermore, there is a lack of effective treatment for GETV infection. Nonetheless, it has not yet received enough attention, and the relevant research about the virus is still insufficient. Therefore, many overwhelming issues, including the immune escape mechanisms of GETV underlying the virus-host interactions, prevention, and treatment, need to be urgently addressed or defined.

IFNs, consisting of classical type I/III IFNs and immune type II IFN (IFN-γ), are important in innate and acquired immunity. Although type I/III IFNs and type II IFNs differ in certain functions and cell/tissue-specific expression, they are all essential drivers of antiviral immunity (Platanias, 2005). Once IFNs bind to their corresponding receptors on the cell surface, numerous IFN-stimulated genes (ISGs) are activated by a signal transduction cascade to produce a plethora of ISG-encoded proteins, which can inhibit viral infection by targeting different phases of the viral replication cycle (Schoggins, 2019). For the genus *Alphavirus*, IFNs have been reported to possess broad-spectrum effective antiviral activity, highlighting the good application potential of IFNs for preventing and controlling those viruses. For example, IFN-γ could reduce viral protein synthesis and inhibit viral RNA transcription of Sindbis virus (SINV) through activating signaling pathways such as Janus kinase-signal transducer and activator of transcription (JAK-STAT) signaling pathway from neurons *in vitro* (Burdeinick-Kerr and Griffin, 2005; Burdeinick-Kerr et al., 2009). Compared with wild-type mice, mice with type I interferon receptor deletion were found to be more susceptible to alphavirus infection and to be even more lethal (Hwang et al., 1995; Ryman et al., 2000). Wild-type adult mice are resistant to the chikungunya virus (CHIKV) infection, while adult mice with a partially or totally abrogated type I IFN pathway develop a mild or severe infection (Couderc et al., 2008; Schilte et al., 2010). However, as a member of the genus *Alphavirus*, whether GETV is susceptible to IFNs and the mechanisms underlying GETV-IFN interaction are poorly reported.

In the current study, the antiviral effect of exogenous IFNs was evaluated *in vitro*, and IFN-γ was demonstrated to have effective anti-GETV activity. Furthermore, the transcriptomic profiling of GETV-infected ST cells was mapped, and possible key factors or pathways involved in the inhibitory effect on virus replication of IFN-γ were preliminarily explored, which indicates that GETV is capable of escaping some innate immune responses, such as IFN-I/III responses, and that IFN-γ could induce biological processes similar to those caused by GETV invasion and other immune processes such as complement and coagulation cascades and the JAK-STAT signaling pathway to inhibit GETV infection. It will contribute to the understanding of GETV-host interaction, provide direction for the study of the molecular mechanism of immune escape, and lay the foundation for the eventual development of drugs against GETV.

## 2. Materials and methods

### 2.1. Cell Lines, antibodies, interferon, and virus preparation

Swine testis (ST) cells were maintained in our laboratory in DMEM (Invitrogen Gibco, United States) supplemented with antibiotics (100 U/mL of penicillin and 100 μg/mL of streptomycin) and 10% fetal bovine serum (FBS) (Invitrogen Gibco, United States) at 37°C with 5% CO_2_. The cell lines present in this study were obtained from Xiamen Immocell Biotechnology Co., Ltd, Guangdong province, China. The mouse anti-GETV-E2 polyclonal antibody was prepared in our laboratory (data not shown); the CF^®^488A goat anti-mouse IgG (H+L) (green) antibody was purchased from Biotium, China; and the HRP-labeled goat anti-mouse IgG antibody was purchased from ZSGB-BIO, China. Porcine interferons were prepared and stocked in our laboratory (Liu et al., 2016). The virus, GETV strain BJ0304 (GenBank accession number OM363683), was stored at our laboratory.

### 2.2. The anti-GETV activity of interferons in ST cells

The ST cells were treated with IFN-α, IFN-ω (referred specifically to as IFN-ω7 in this study), IFN-γ, IFN-λ3, or PBS for 24 hours (h) either 24 h before or 0 and 12 h post-infection (hpi) with GETV [multiplicity of infection (MOI) = 0.1], and mock-infected cells were used as controls. At the indicated time points after infection, after observing and recording the cytopathic effects (CPEs), the cell samples and the supernatant were collected. The former was used to analyze the expression levels of E2 by immunological methods (i.e., IFA and Western blot) and to detect the copies of GETV *nsp3* (Supplementary Table S1) by RT-qPCR, while the supernatant samples were used for the TCID_50_ assay according to the Reed and Muench method (Reed and Muench, 1938).

### 2.3. Indirect immunofluorescence assay (IFA)

ST cell monolayers with or without IFN pro-treatment were inoculated with GETV at an MOI of 0.1 for 1.5 h and then cultured with DMEM with 2% FBS for the indicated time points after infection before analyzing the GETV infection using IFA as described by Li et al. (2017).

### 2.4. RT-qPCR

The IFN-γ-treated and/or GETV-infected and mock-infected ST cells were collected for RNA extraction using TRIzol reagent (Invitrogen, America), followed by cDNA synthesis. The GETV *nsp3* and some genes selected from statistical analysis of RNA-seq were determined by RT-qPCR using the ChamQ Universal SYBR qPCR Master Mix (Vazyme, China), and *GAPDH* was used as an endogenous control. All primers used are listed in Supplementary Table S1.

### 2.5. Illumina RNA-Seq library preparation, deep sequencing, and sequence analysis

Mock- and GETV-infected (MOI=0.1) ST cells with or without IFN-γ were collected at 20 hpi for RNA-sequencing. Then, 12 samples consisting of three repetitions in each group were used in this study. Illumina RNA-Seq library preparation, deep sequencing, and sequence analysis were performed as described by Liu et al. (2020). mRNA was selected using oligo(dT)-attached magnetic beads from purified total RNA. After treatment with fragmentation buffer, approximately 300 bp mRNA fragments were selected and converted to cDNA. Adaptors were ligated to the ends of the cDNA fragments before end-repair and dA-tailing and then amplified using an Illumina TruseqTM RNA sample Prep Kit. Libraries sequencing was carried out on an Illumina Novaseq 6000 Sequencing System, completed by Meggie Biotechnology Co., Ltd.

The raw data (reads) obtained from the Illumina Novaseq 6000 Sequencing System were processed and counted by fastp to obtain the clean data (reads), followed by being mapped to the *Sus scrofa* genome (RefSeq GCF_000003025.6, https://www.ncbi.nlm.nih.gov/genome/?term=Sus) to obtain the mapped data (reads) using the HISAT2 (v2.1.0) program with default parameters. To identify differentially expressed genes (DEGs) between two different groups, the expression level of each transcript was calculated according to the transcripts per million reads (TPM) method. DESeq2 (version 1.24.0) with parameters including *P*_adjust_ < 0.05 and |log2FC|≥1 was used to perform DEG analysis before gene expression quantitative analysis was carried out using the RSEM (v1.3.3) program. The DEGs were further analyzed by Gene Ontology (GO) analysis and Kyoto Encyclopedia of Genes and Genomes (KEGG) enrichment analysis, which were performed using the GOATOOLS (v0.6.5) program and R script with adjusted *P*_adjust_ < 0.05, respectively. The *P*-value was calculated using Fisher's exact test. The heatmap analysis of DEGs was conducted with ClustVis (https://biit.cs.ut.ee/clustvis/). The Venn diagram of DEGs among pairwise comparisons was performed in imageGP (https://www.bic.ac.cn/ImageGP/index.php/Home/Index/VennDiagram.html). Moreover, protein–protein interaction (PPI) network analysis was performed using the STRING server (https://cn.string-db.org/cgi/input?sessionId=bYlaUtwqKXnr&input_page_show_search=on). The raw data were submitted to the Sequence Read Archive (SRA) database (accession: PRJNA799863).

### 2.6. Statistics analysis

The data were analyzed using GraphPad Prism 8. Comparisons were determined using a two-way ANOVA. Differences were considered significant if the *P* < 0.05. *P*-values are indicated as follows: ns > 0.05; ^*^P < 0.05; ^**^P < 0.01; ^***^P < 0.001; ^****^P < 0.0001.

## 3. Results

### 3.1. GETV was susceptible to IFN-γ in ST cells

**Figure 1:**
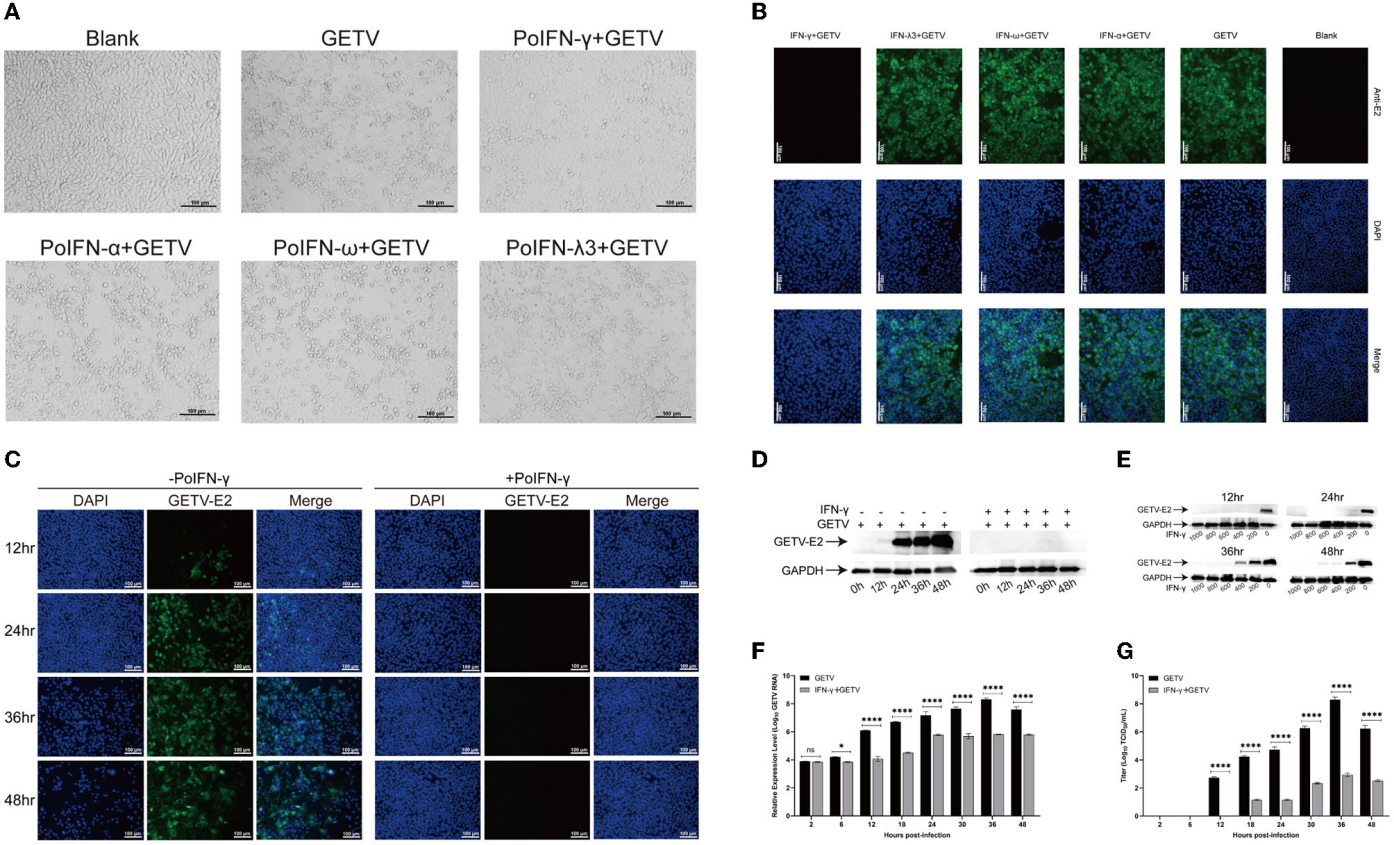
Anti-Getah virus (GETV) activity of interferons *in vitro*. **(A)** Virus sensitivity to interferon (IFN) treatment (cytopathic effects (CPE) inhibition assay). The ST cells were treated with IFN-α, IFN-ω, IFN-γ, IFN-λ3, or PBS for 24 h before being infected with GETV (multiplicity of infection (MOI)=0.1), and mock-infected cells were used as controls. At 48 hpi, the cells were visualized using an ECLIPSE Ti-S fluorescence microscope. Scale bars represent 100 μm. **(B)** At 24 hpi, an indirect immunofluorescence assay (IFA) was used to detect the expression level of GETV E2 in GETV-infected cells with different interferon pre-treatments. Nuclei were visualized by staining with DAPI. Scale bars represent 100 μm. **(C)** At different hours post-infection, IFA was used to detect the expression level of GETV E2 in GETV-infected cells with IFN-γ pre-treatment. Nuclei were visualized by staining with DAPI. Scale bars represent 100 μm. **(D)** At different hours post-infection, the reduction of GETV E2 expression level in infected cells with a high dose of IFN-γ pre-treatment. **(E)** The reduction of GETV E2 expression level in infected cells with different doses of IFN-γ pre-treatment (0, 200, 400, 600, 800, and 1,000 IU/mL). The infected ST cells were incubated with a mouse anti-GETV-E2 polyclonal antibody and an HRP-labeled goat anti-mouse IgG antibody. **(F, G)** At different hours post-infection, the reduction of RNA copy number and titer of GETV in infected cells with/without IFN-γ pre-treatment. The results are presented as the mean ± SD (*N* = 3). *P*-values are indicated as follows: ns > 0.05; ^*^P < 0.05; ^**^P < 0.01; ^***^P < 0.001; ^****^P < 0.0001.

To determine the replication efficiency of GETV treated with different doses of IFN-α, IFN-ω, IFN-γ, and IFN-λ3, ST cells were infected with GETV (MOI=0.1). Among the three different treatment methods, pre-treatment with IFNs showed the best inhibitory effect on viral replication (data not shown) and was selected for further research. An immunofluorescence assay (IFA) and cytopathic effect (CPE) assay showed that pre-treatment with a high dose (1,000 IU/mL) of recombinant porcine IFN-γ had a significant inhibitory effect on GETV replication in ST cells. In contrast, viral proliferation was not significantly affected by IFN-I (IFN-α, ω) and IFN-III (IFN-λ3), even with a high dose (1,000 IU/mL), as determined through IFA and CPE assays (Figures 1A, B). To verify the antiviral property of IFN-γ against GETV, the copy number of the virus, viral titer, and the expression level of structural protein E2 at different time points under the condition of a single (1,000 IU/mL) or gradient dose (from 1,000 IU/mL to 200 IU/mL) of IFN-γ pre-treatment were detected. As shown in Figure 1F, at 6–48 hpi, the transcriptional level of GETV in GETV-infected ST cells treated with the high dose (1,000 IU/mL) of IFN-γ was significantly lower than that in GETV-infected ST cells without IFN-γ treatment. The suppression effect on the virus titer of GETV was similar to the transcriptional level and appeared to be more remarkable at 12–48 hpi (Figure 1G). IFA and WB assays showed that pre-treatment with a high dose (1,000 IU/mL) of IFN-γ almost completely inhibited the translation level of GETV E2 at 0–48 hpi (Figures 1C, D). These results indicated that the high dose of IFN-γ could exert an obvious suppression on the transcriptional level of GETV, virus titer, and the expression level of the structural protein E2. By comparing the results between Figures 1D, F, almost no protein E2 was detected during 0–48hpi, indicating that the inhibitory effect of IFN-γ on the translation level appears to be stronger than on the transcriptional level if the sensitivity biases between qRT-PCR and WB were not taken into account. Moreover, the expression of E2 could be inhibited by IFN-γ in a dose-dependent manner in the range of 0–800 IU/mL, and complete inhibition could be obtained at all observed time points when the dose is up to 800 IU/mL (Figure 1E). These data may indicate that GETV has evolved mechanisms to antagonize the IFN-I/III responses by targeting the IFN-induced downstream instead of its induction signaling pathway. Furthermore, pre-treatment with IFN-γ could suppress GETV replication, and the inhibiting effect lasted for at least 48 h.

### 3.2. The landscape of differentially expressed genes (DEGs) identified in pairwise comparisons

**Figure 2:**
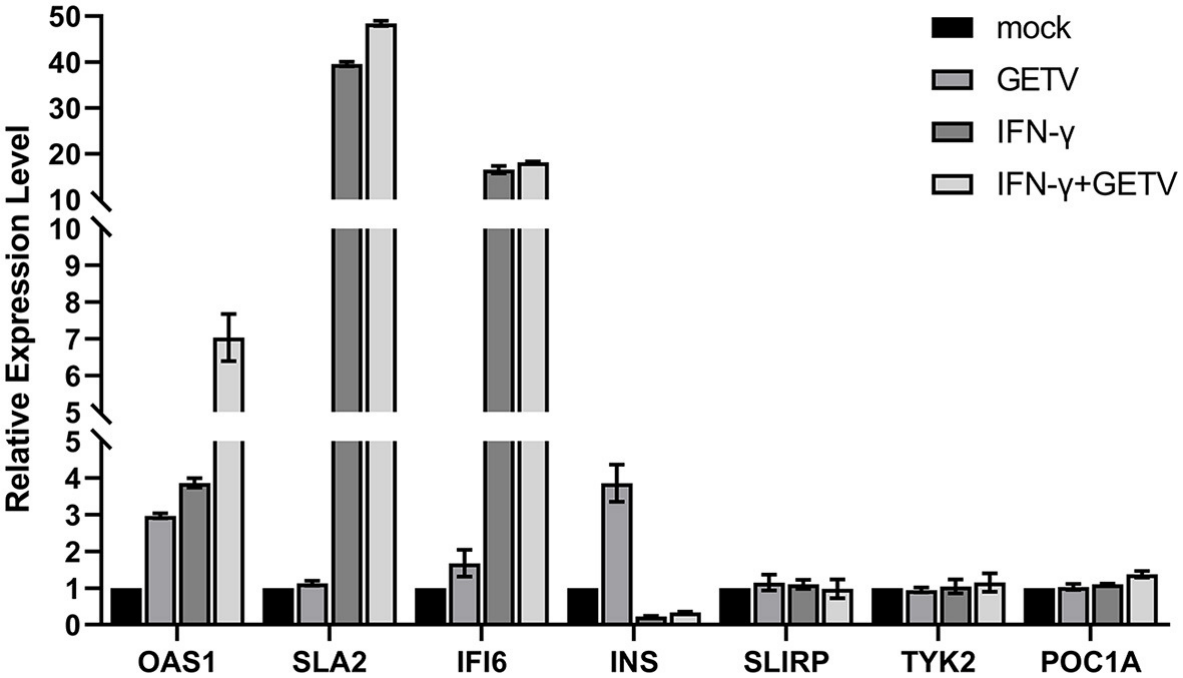
Validation of RNA-Seq Data by qPCR. Seven genes were randomly selected and simultaneously verified by RT-qPCR. The results are presented as the mean ± SD (N = 3).

The mRNA-transcriptional levels of mock- and GETV-infected ST cells with or without IFN-γ pre-treatment were determined by high-throughput sequencing. DEGs in host cells from four groups were identified based on a defined criteria (fold change > 2.0 or < 0.5; p_adjust_ < 0.05). To validate mRNA-transcriptional levels obtained from RNA-Seq, seven genes were randomly selected and simultaneously verified using RT-qPCR. The results showed good correlations of all genes between assays (Figure 2), indicating the accuracy and validity of the RNA-Seq data, which could be used for further analysis, such as GO, KEGG, heatmap, and STRING analyses.

**Figure 3:**
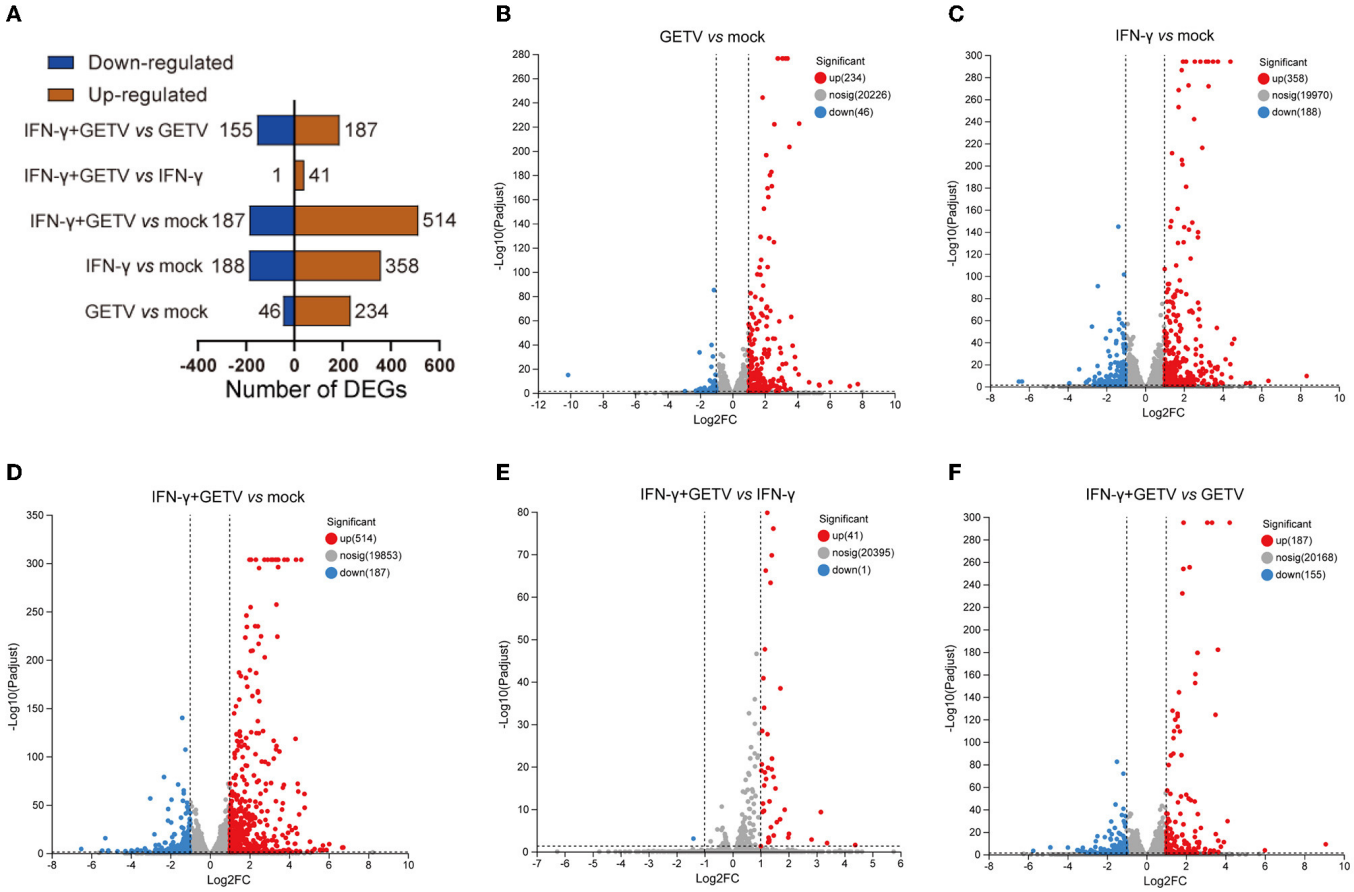
The landscape of differentially expressed genes (DEGs) identified in pairwise comparisons. Based on the pairwise comparisons of mock, GETV, IFN-γ, and IFN-γ+GETV, **(A)** the number of DEGs in ST cells was summarized, and volcano plots for DEGs in ST cells from pairwise comparisons of GETV *vs*. mock **(B)**, IFN-γ *vs*. mock **(C)**, IFN-γ+GETV *vs*. mock **(D)**, IFN-γ+GETV *vs*. IFN-γ **(E)** and IFN-γ+GETV *vs*. GETV **(F)** were drawn.

In pairwise comparisons, 280 DEGs (234 upregulated and 46 downregulated) in GETV *vs*. mock, 546 DEGs (358 upregulated and 188 downregulated) in IFN-γ *vs*. mock, 701 DEGs (514 upregulated and 187 downregulated) in IFN-γ+GETV *vs*. mock, 42 DEGs (41 upregulated and 1 downregulated) in IFN-γ+GETV *vs*. IFN-γ, and 342 DEGs (187 upregulated and 155 downregulated) in IFN-γ+GETV *vs*. GETV were identified, respectively (Figure 3A). Volcano plots were also drawn (Figures 3B–F). Using this approach, we visualized the transcriptional levels and statistical significance of all genes among groups. Meanwhile, by mapping the clean data to the GETV BJ0304 genome, few clean data in IFN-γ+GETV could be mapped to the GETV genome (Table 1), indicating the good inhibitory effect of IFN-γ on GETV at 20 hpi, which further attested the results observed in Figure 1.

**Table 1:** Transcriptional level of the GETV genome in ST cells at 20 hpi.

**GETV-1**	**GETV-2**	**GETV-3**	**IFNγ+GETV-1**	**IFNγ+GETV-2**	**IFNγ+GETV-3**
36.17%	36.25%	16.06%	0.00%	0.00%	0.00%

Data are presented as the percentage of the sum of the clean data mapped to the GETV genome in the total clean data.

### 3.3. Gene ontology (GO) and Kyoto Encyclopedia of Genes and Genomes (KEGG) analyses of DEGs in pairwise comparisons

**Figure 4:**
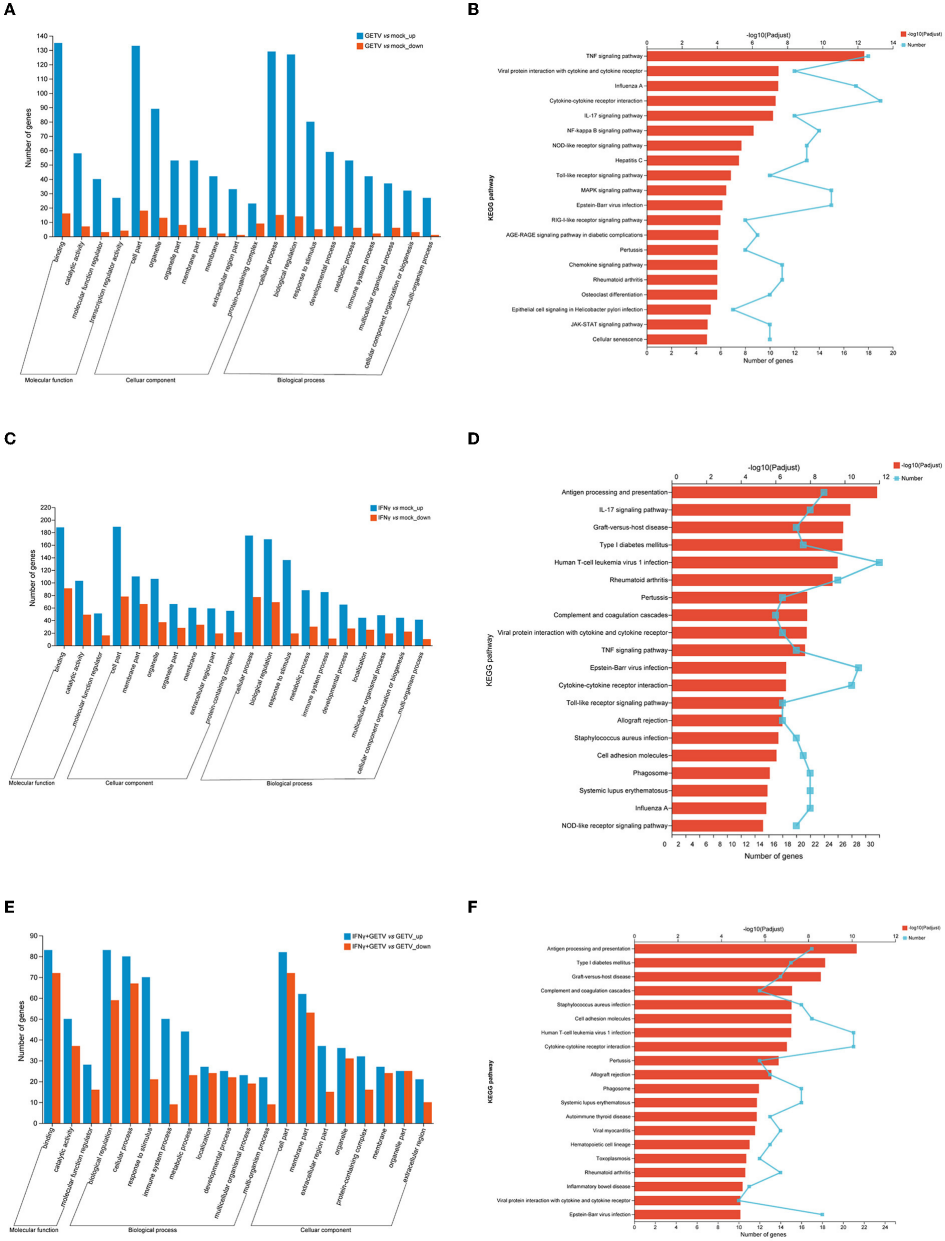
Gene ontology (GO) and Kyoto Encyclopedia of Genes and Genomes (KEGG) analysis of differentially expressed genes (DEGs) in pairwise comparisons. **(A)** GO analyses of differentially expressed genes in GETV relative to the mock (GETV *vs*. mock). **(B)** KEGG analyses of differentially expressed genes in GETV *vs*. mock. **(C)** GO analyses of differentially expressed genes in IFN-γ relative to the mock (IFN-γ *vs*. mock). **(D)** KEGG analyses of differentially expressed genes in IFN-γ *vs*. mock. **(E)** GO analyses of differentially expressed genes in GETV treated with IFN-γ relative to GETV (IFN-γ+GETV *vs*. GETV). **(F)** KEGG analyses of differentially expressed genes in IFN-γ+GETV *vs*. GETV. DEGs were identified based on defined criteria (fold change>2.0 or <0.5; p < 0.05). Blue column: upregulated genes; red column: downregulated genes **(A, C, E)**. Broken lines indicate the number of genes, and columns depict the significance level **(B, D, F)**.

To obtain systematic insights into the host responses to GETV infection and/or IFN-γ stimulation, DEGs in GETV *vs*. mock, IFN-γ *vs*. mock, and IFN-γ+GETV *vs*. GETV were selected and analyzed using the GO and KEGG databases. GO analysis of GETV *vs*. mock indicated that most of the identified DEGs were involved in cellular processes, biological regulation, and the response to stimuli; were located in the cell part, organelle, and organelle parts; and possessed binding, catalytic activity, and molecular function regulators (Figure 4A). GO analyses of both IFN-γ *vs*. mock and IFN-γ+GETV *vs*. GETV showed that the top three enriched cellular components, molecular function, and biological process were almost identical to the corresponding terms of GETV *vs*. mock (Figures 4A, C, E).

The 20 most significantly enriched pathways of the three pairwise comparisons revealed broad changes in the different biological states. At 20 hpi, DEGs were abundant in host pattern recognition receptor (PRR)-related pathways (i.e., NOD-like receptors (NLRs) (Supplementary Table S2), toll-like receptor (TLR) (Supplementary Table S3), and RIG-I-like receptor (RLR) (Supplementary Table S4) signaling pathway and its downstream pathways, including the NF-kappa B signaling pathway (Supplementary Table S5), the tumor necrosis factor (TNF) signaling pathway (Supplementary Table S6), the IL-17 signaling pathway (Supplementary Table S7), viral protein interaction with cytokine and cytokine receptor (Supplementary Table S8), cytokine-cytokine receptor interaction (Supplementary Table S9), the chemokine signaling pathway (Supplementary Table S10), and the JAK-STAT signaling pathway (Supplementary Table S11). Meanwhile, pathways involved in cell proliferation and differentiation, such as the mitogen-activated protein kinase (MAPK) signaling pathway (Supplementary Table S12) and cellular senescence (Supplementary Table S13), were also significantly affected by GETV infection (Figure 4B). The most significantly enriched signaling pathway of IFN-γ+GETV *vs*. GETV was antigen processing and presentation (Supplementary Table S14), which was followed by some intriguing pathways such as complement and coagulation cascades (Supplementary Table S15), cell adhesion molecules (Supplementary Table S16), cytokine-cytokine receptor interaction (Supplementary Table S17), and phagosome (Supplementary Table S18), all of which were overlapped in the IFN-γ vs. mock (Supplementary Tables S19–S23). This result indicated that the basic and vital pathways of the host induced by IFN-γ could not be changed by GETV (Figures 4D, F). In addition, IL-17 (Supplementary Table S24), TNF (Supplementary Table S25), and TLR signaling pathways (Supplementary Table S26) were identified in IFN-γ *vs*. mock (Figure 4D), while hematopoietic cell lineage (Supplementary Table S27) was activated in IFN-γ+GETV *vs*. GETV (Figure 4F).

### 3.4. Heatmap analysis of DEGs in pairwise comparisons

To map the transcriptomic profiling of GETV-infected ST cells and preliminarily explore possible key factors or pathways involved in the inhibitory effect on the virus replication of IFN-γ, intriguing DEGs above in GETV *vs*. mock and IFN-γ+GETV *vs*. GETV were selected and analyzed.

**Figure 5:**
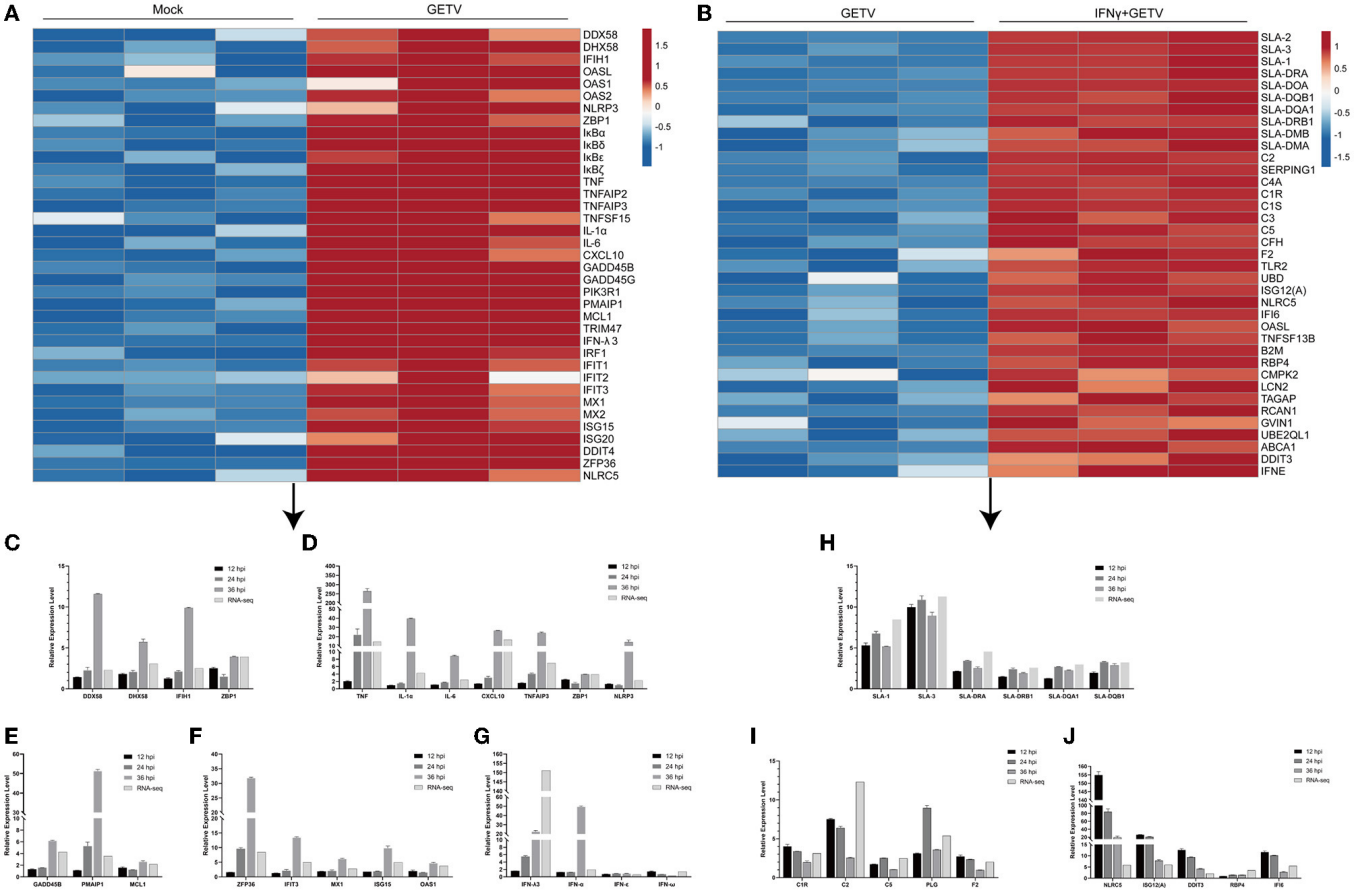
A heatmap analysis of DEGs in pairwise comparisons. **(A, B)** The heatmap displays the transcriptional level of some intriguing DEGs in GETV *vs*. mock **(A)** and IFN+GETV *vs*. GETV **(B)**. The data are normalized Transcripts Per Million reads (TPM) values. The color scale is shown at the right of the heatmap, and red boxes indicate higher levels of expression, while blue boxes indicate lower levels of expression. **(C–G)** Regulation of gene expression in ST cells infected with GETV at a MOI of 0.1 for the indicated time points. **(C)** Transcriptional level of select DEGs in PRRs-related pathways at different time points in GETV *vs*. mock. **(D)** Transcriptional level of select DEGs among inflammatory and cytokine-related factors at different time points in GETV *vs*. mock. **(E)** Transcriptional level of select DEGs among apoptotic factors at different time points in GETV *vs*. mock. **(F)** Transcriptional level of select DEGs among antiviral interferon-stimulated genes (ISGs) at different time points in GETV *vs*. mock. **(G)** Transcriptional level of interferons (IFNs) at different time points in GETV *vs*. mock. **(H–J)** Regulation of gene expression in infected ST cells treated with IFN-γ. **(H)** Transcriptional level of select DEGs in the antigen processing and presentation pathway at different time points in IFN-γ+GETV *vs*. GETV. **(I)** Transcriptional level of select DEGs in complement and coagulation cascades at different time points in IFN-γ+GETV *vs*. GETV. **(J)** Transcriptional level of select DEGs among ISGs at different time points in IFN-γ+GETV *vs*. GETV. Data are presented as the mean ± SD (*N* = 3).

After GETV infection, a large number of genes involved in the RLR signaling pathway, the NLR signaling pathway, the cytokine-related pathways, and the IFN-response signaling pathway were identified as DEGs and upregulated, some of which were validated by RT-qPCR (Figures 5A, C, D, F, G). Besides, some DEGs (*GADD45B, GADD45G, PIK3R1, PMAIP1, MCL1, TNF*, and *NFKBIA*) were enriched in the apoptotic pathway (Figures 5A, E). Furthermore, the results of RT-qPCR showed that GETV infection could upregulate the transcriptional levels of IFN-λ3, as well as IFN-α, despite the existence of a temporal difference (Figure 5G). By combining the results of the antiviral assay (Figures 1A, B), it could be speculated that GETV has evolved the mechanisms to antagonize the IFN-I/III responses by targeting the IFNs induced downstream instead of its induction signaling pathways.

With the pre-treatment of IFN-γ, numerous genes are involved in the antigen processing and presentation pathway (e.g., *SLA-1, SLA-2, SLA-3, SLA-DRA, SLA-DRB1, SLA-DQA1, SLA-DQB1, SLA-DOA, SLA-DMA*, and *SLA-DMB*) and complement and coagulation cascades (e.g., *C2, C3, C5, C1R, C1S, C4A, CFH*, and *F2*). However, some important antiviral ISGs (*ISG12(A), NLRC5, IFI6, OASL, RBP4*, and *DDIT3*) were significantly upregulated in GETV-infected ST cells (Figure 5B). Based on the bioinformatics analysis and the fold changes in mRNA level, 16 genes were selected and verified by RT-qPCR (Figures 5H–J).

### 3.5. Combined analysis among pairwise comparisons

After the analysis of each pairwise comparison, the combined analyses were carried out to further understand how IFN-γ inhibits the replication of GETV.

#### 3.5.1. IFN-γ could exert biological processes analogous to those induced by GETV

**Figure 6:**
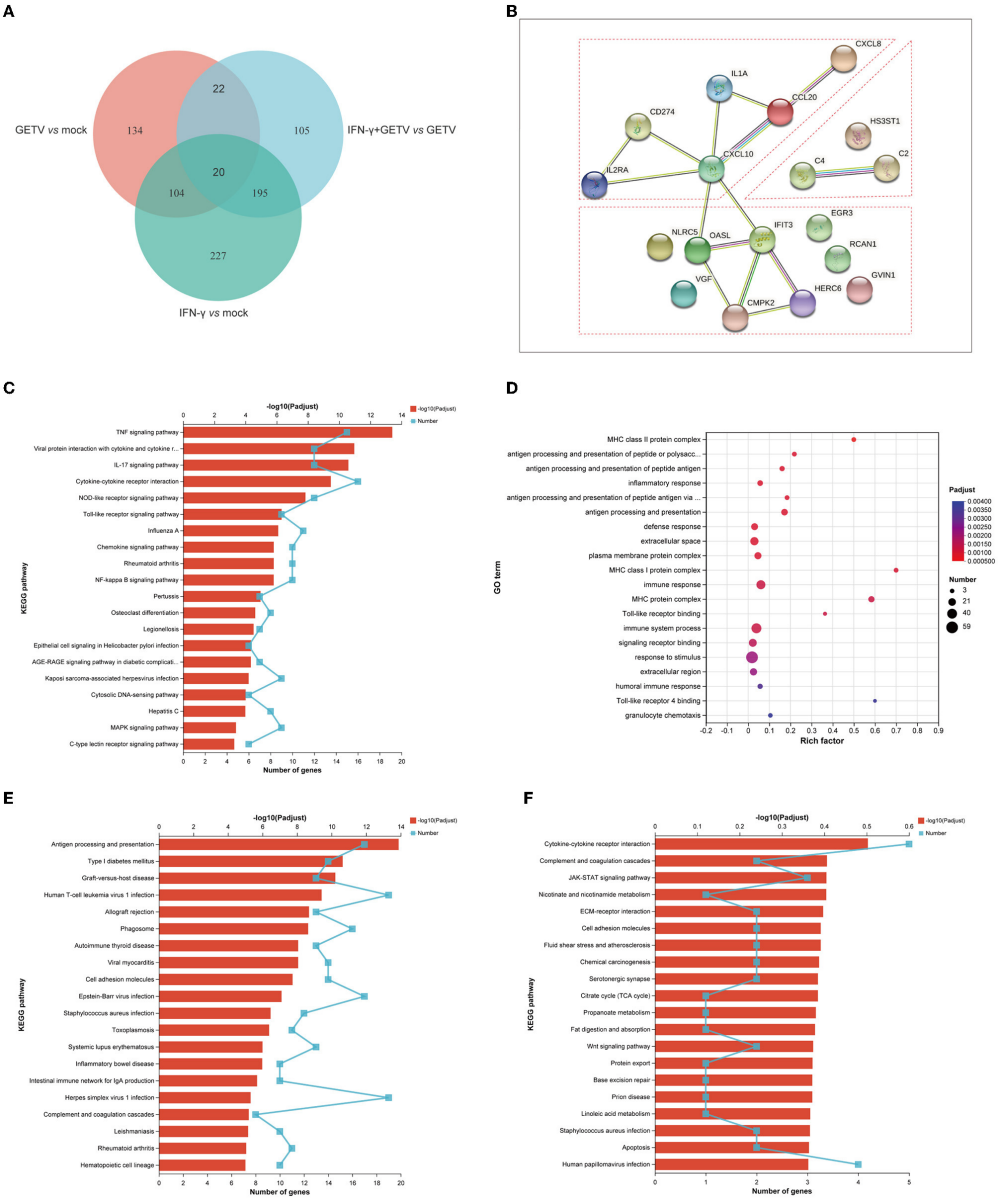
Combined analysis among pairwise comparisons. **(A)** A Venn diagram analysis of DEGs among GETV vs. mock, IFN-γ vs. mock, and IFN-γ + GETV vs. GETV. **(B)** Protein-Protein Interaction Networks (PPI) of common 20 DEGs among GETV vs. mock, IFN-γ vs. mock, and IFN-γ + GETV vs. GETV. **(C)** KEGG analyses of 124 DEGs shared in pairwise comparisons of GETV *vs*. mock and IFN-γ *vs*. mock. **(D)** GO and **(E)** KEGG analyses of 195 DEGs shared in pairwise comparisons of IFN-γ *vs*. mock and IFN-γ+GETV *vs*. GETV. **(F)** KEGG analyses of 105 DEGs are presented in IFN-γ+GETV *vs*. GETV but not in the other two groups.

The analysis of 20 DEGs is shown in a Venn diagram showing pairwise comparisons (Figure 6A) and the DEGs were clustered in cytokine-related factors, ISGs, and complements by STRING analysis (Figure 6B). Intriguingly, almost all 20 DEGs were upregulated in all three pairwise comparisons (Table 2). These results might suggest that the activation of the 20 DEGs may play a fundamental and crucial role in an anti-GETV signaling pathway.

**Table 2:** The common 20 DEGs from Venn diagram analysis among three pairwise comparisons.

**Gene name**	**GETV vs. mock**	**IFN-γ vs. mock**	**IFN-γ+GETV vs. GETV**
C2	Up	Up	Up
C4A	Up	Up	Up
CCL20	Up	Up	Up
CD274	Up	Up	Up
CMPK2	Up	Up	Up
CXCL10	Up	Up	Down
CXCL8	Up	Up	Up
EGR3	Up	Up	Up
GVIN1	Up	Up	Up
HERC6	Up	Up	Up
HS3ST1	Up	Up	Up
IFIT3	Up	Up	Down
IL1A	Up	Up	Up
IL2RA	Up	Up	Up
LOC110255361	Up	Up	Up
NLRC5	Up	Up	Up
OASL	Up	Up	Up
RCAN1	Up	Up	Up
SULT1E1	Down	Down	Down
VGF	Up	Up	Up

To further investigate the common effects of GETV and IFN-γ on host cells, 124 DEGs shared in pairwise comparisons of GETV *vs*. mock and IFN-γ *vs*. mock were analyzed. The results found that the 124 DEGs showed the same trend in the two groups (Supplementary Figure S1), indicating that IFN-γ could induce biological processes similar to those caused by GETV invasions, such as immune responses and antiviral responses (Figure 6C), as well as to play an antiviral role, which appeared to support the results of the above 20 DEGs.

#### 3.5.2. IFN-γ could induce a specific immune process to inhibit the replication of GETV

The potent anti-GETV effect appears not to be solely achieved by the biological processes simultaneously induced by both IFN-γ stimulation and GETV infection, which may be related to other biological processes specifically induced by IFN-γ stimulation. Therefore, 195 DEGs shared in pairwise comparisons of IFN-γ *vs*. mock and IFN-γ+GETV *vs*. GETV were analyzed, and the results indicated that most of them are involved in immune system processes such as antigen processing and presentation, defense response, and complement and coagulation cascades (Figures 6D, E). Besides, the up-down trend of the 195 DEGs was completely consistent between IFN-γ vs. mock and IFN-γ+GETV vs. GETV (Supplementary Figure S2), and their count of fold change is similar (Supplementary Figure S2), which indicated that the expression pattern of these antiviral genes induced by IFN-γ may not be significantly affected by GETV infection, suggesting that they may be the major force for IFN-γ to inhibit the replication of GETV. Furthermore, 105 DEGs presented in IFN-γ+GETV *vs*. GETV were also investigated by bioinformatic analysis and were abundant in cytokine-cytokine receptor interaction, complement and coagulation cascades, JAK-STAT signaling pathways, and other immune-related pathways (Figure 6F). Interestingly, the genes involved in the immune-related pathways among the 105 DEGs were almost upregulated in IFN-γ+GETV *vs*. GETV (Supplementary Figure S3), while other downregulated DEGs were significantly enriched in biological processes, including metabolic and developmental processes (Supplementary Table S28).

## 4. Discussion

GETV, a mosquito-borne arbovirus with a wide host range and broad geographical distribution, appears to be an increasingly definite threat to the global community, especially the Chinese livestock and poultry industries, and even to public health (Lu et al., 2020). Currently, awareness and attention to the virus are still inadequate, emphasizing the urgency and necessity of conducting associated research to prevent and control the virus.

### 4.1. Specific host PRRs and their downstream pathways are activated in ST cells exposed to GETV

PRRs are predominantly expressed in innate immune cells, such as dendritic cells (DCs) and macrophages, as well as non-immune cells, such as fibroblast cells and epithelial cells. Host PRRs include a special domain such as the ectodomain with leucine-rich repeats (LRRs), which could recognize viral proteins or nucleic acid components termed pathogen-related molecular patterns (PAMPs), thereby initiating intracellular signal transduction through the adaptor protein (STING, MAVS, MyD88) and finally activating the innate immune response to exert an antiviral effect (Thompson et al., 2011; Kawasaki and Kawai, 2014). Nevertheless, few studies about GETV have focused on this issue, and the mechanism of host-GETV interaction remains unknown. In this study, an in-depth insight into the transcriptional changes of GETV-infected ST cells was obtained using RNA-seq, which would help us to explore mechanisms underlying the host-pathogen interaction. Using RNA-seq, 280 DEGs in total, including 234 upregulated and 46 downregulated genes, were identified (Figures 3A, B), and the main PRRs and core genes involved in compelling pathways such as the cytokine-, apoptosis-, and antiviral-related pathways screened by comprehensive analyses (Figures 4B, 5A) triggered our interest.

Among the PRRs, RLRs, including three cytoplasmic RNA helicases (i.e., DDX58/RIG-I, IFIH1/MDA-5, and DHX58/LGP2), are essential for sensing viral RNA and initiating the innate immune response against virus infection. Both DDX58 and IFIH1 are composed of two N-terminal caspase recruitment domains (CARDs), a central DEAD-box helicase/ATPase domain, and a C-terminal regulatory domain (RD). Unlike them, DHX58 lacks the CARD, containing only the helicase domain and RD (Yoneyama et al., 2005; Takeuchi and Akira, 2009). DDX58 and IFIH1 recognize different RNA viruses by detecting short dsRNAs (up to 1kb) with 5′ triphosphate ends and long dsRNAs (>2 kb), respectively (Takeuchi and Akira, 2010). Studies have shown that DDX58 is able to recognize hepatitis C virus (HCV), vesicular stomatitis virus (VSV), Japanese encephalitis virus (JEV), Sendai virus (SV), and influenza virus. IFIH1 can recognize encephalomyocarditis virus (EMCV), mengo virus, Semliki Forest virus (SFV), and poliovirus (Kato et al., 2006; Saito et al., 2008; Pichlmair et al., 2009; Linder et al., 2021). DDX58 and IFIH1 can interact with IPS-1, which then activates signaling cascades leading to the expression of type I IFN genes via EYA4, TRAF3, NAP1/SINTBAD, TBK1/IKK-i, and IRF3/7. DHX58 functions as a positive regulator in RIG-I-mediated and MDA5-mediated virus recognition (Takeuchi and Akira, 2010). In this study, all members of *RLRs* were significantly upregulated in RNA-seq as expected and in further confirmation at different time points (Figures 5A, C); however, some important dsRNA/ssRNA sensors (e.g., *TLR3* and *TLR7*) were not identified as DEGs. TLR3, a member of the TLR family proteins that is present in the endosome and recognizes dsRNA, can induce type I IFNs via the adaptor protein TIR-domain-containing adapter inducing IFN-β (TRIF) (Matsumoto and Seya, 2008). TLR7 is essential for viral ssRNA-induced type I IFN production (Kato et al., 2005; Takeuchi and Akira, 2010). Scientists are still split about the concept that ZBP1 (DAI) is a dsDNA sensor. Although no dsDNA occurred in the life cycle of GETV as an ssRNA virus, *ZBP1* was also identified and significantly upregulated (Figures 5A, C), which may, through recognizing Z-RNA (Jiao et al., 2020), activate the downstream signaling pathway.

In this study, over 15 cytokines/chemokines were upregulated in response to GETV infection. TNF is a key mediator and regulator of immune responses, mainly via the induction of NF-κB and MAP pro-survival kinases and activation of cell death in certain pathological situations to exert its biochemical functions (Varfolomeev and Vucic, 2018). The transcriptional level of *TNF* was upregulated in a time-dependent manner after GETV infection (Figures 5A, D). Besides, some other important pro-inflammatory cytokines, *IL-6* and *IL-1*α, as well as *NLRP3* (NOD-like receptor protein 3), were upregulated to a top degree at 36 hpi (Figures 5A, D), indicating a higher inflammation response for viral clearance at the late phase after infection, which was supported by the report that the expression level of NLRP3 was positively correlated with the peak inflammatory symptoms (Chen et al., 2017). Alphavirus is well known for its capability to trigger the host cell to activate its genetically programmed cell death pathway, leading to the morphological features of apoptosis (Griffin and Hardwick, 1997; Dhanwani et al., 2012; Wang et al., 2012). However, as a member of the genus *Alphavirus*, this issue about GETV remains poorly reported. In this study, *GADD45B*, a promoter of apoptosis, was gradually upregulated over time after GETV infection, and a largely consistent situation was also observed in *NOXA* (*PMAIP1*) and *MCL1* (Figures 5A, E). However, the MCL1/NOXA axis is involved in the intrinsic pathway of apoptosis, where NOXA selectively binds to MCL1 and prevents it from inhibiting apoptosis. Thus, it could be speculated that GETV may regulate host apoptosis in multiple ways or in some feedback manners. The cellular mechanisms involved in GETV-induced apoptosis are complex, and much information needs to be further explored.

Furthermore, induction of IFN-α and IFN-λ3 (Figures 5A, G) and activation of downstream antiviral responses with a series of upregulated ISGs that limit viral proliferation were observed after infection (Figures 5A, F). Among the ISGs, the OAS family could activate the latent ribonuclease (RNase L) to degrade basal mRNAs (Floyd-Smith et al., 1981) and escape the antiviral mRNAs from Rnase L-mediated mRNA decay (Burke et al., 2019), while Mx1 could block the entry of viral nucleic acids into the cell (Nigg and Pavlovic, 2015; Verhelst et al., 2017). Interestingly, Mx1 can also inhibit the production of type I IFN by regulating the phosphorylation of IRF3 in a negative-feedback manner (Schattgen et al., 2016). Thus, it could be speculated that GETV may evolve multiple mechanisms to enable the evasion of IFN-related immune responses.

Although GETV infection boosted inflammatory factors and ISG expression to achieve an antiviral niche or milieu in ST cells, it appears that this antiviral state could affect GETV proliferation only with low magnitude (Figures 1A, B). Meanwhile, viruses have evolved multiple mechanisms to enable evasion of host immune response, such as the IFN system in the process of virus-host interaction (Garcia-Sastre, 2017; Nelemans and Kikkert, 2019), which also occurred in alphaviruses mainly via nsP2, including the examples that CHIKV disables the unfolded protein response of the host (Fros et al., 2015) and that SINV inhibits IFN production (Frolova et al., 2002) by targeting STAT signaling directly (Fros et al., 2016). Following almost non-effective endogenous IFN-α/λ3 (Figures 5A, G), exogenous IFN-α/ω and IFN-λ3 pre-treatment with high biological activity failed to inhibit the viral infection and early replication *in vitro* (Figures 1A, B). It could be speculated that GETV may have developed strategies that avoid the action of IFN by preventing the binding of viral products to cellular sensors and by inactivating downstream cellular factors involved in IFN signal transduction or the establishment of the antiviral state because the depths of anti-virus ISG and other immune systems, such as complement and coagulation cascades, are limited in the host infected by GETV, indicating that GETV enables the evasion of host immunity through some uncertain mechanisms.

### 4.2. Exogenous IFN-γ can inhibit GETV replication *in vitro*

IFN-γ is a pleiotropic cytokine that has immunomodulatory effects, including enhancement of NK- and T-cell-mediated cytotoxicity, B-cell differentiation, surface antigen expression, and macrophage activation, as well as antiviral effects through partially overlapping but distinct signaling cascades with type I/III IFNs (Ramana et al., 2002). Although IFN-γ is an immune cell-confined type II IFN, its receptors (IFNGR1/2) are widely distributed in almost all cell types (Fan et al., 2020), including ST cells (Shan et al., 2019). As a broad-spectrum antiviral agent, besides human viruses such as the Ebola virus (Rhein et al., 2015), Zika virus (Chaudhary et al., 2017), and antiviral-resistant hepatitis C virus (Meissner et al., 2010), IFN-γ has been shown to enable the direct inhibition of numerous porcine viruses, such as the porcine reproductive and respiratory syndrome virus (PRRSV) (Hamana et al., 2017), foot-and-mouth disease virus (FDMV) (Bautista and Molitor, 1999), and African swine fever virus (ASFV) (Fan et al., 2020), indicating significant application potential prospects in the therapeutic or preventive field, which also extends to GETV for the first time in our study. Contrary to IFN-α/λ3/IFN-ω, IFN-γ presented effective inhibitory activity against GETV in a dose-dependent manner in ST cells in this study, and the inhibiting effect did not weaken significantly over a long period (Figure 1), which may lead to the development of novel strategies for the treatment of GETV.

A total of 342 DEGs (187 upregulated and 155 downregulated) between GETV and GETV treated with IFN-γ were identified (Figure 3A). Among them, KEGG and heatmap analysis combined with RT-qPCR showed that, besides antigen processing and presentation (Figures 4F, 5B, H), complement and coagulation cascades (Figures 4F, 5B, I) coupled with some ISGs such as *NLRC5, OASL, ISG12A, DDIT3, RBP4*, and *IFI6* (Figures 5B, J) were activated in response to IFN-γ treatment. Moreover, the combined analysis among pairwise comparisons of GETV *vs*. mock, IFN-γ *vs*. mock, and IFN-γ+GETV *vs*. GETV was performed to further investigate how IFN-γ inhibits the replication of GETV. A total of 20 DEGs were shared among groups (Figure 6A) and clustered in cytokine-related factors, ISGs, and complements (Figure 6B). These results revealed that the activation of complement and coagulation cascades, coupled with some important ISGs, may play synergetic roles in the anti-GETV activity of IFN-γ. Intriguingly, NLRC5, a specific IFN-γ-induced atypical NLR family member that is essential for the IFN-γ-induced activation of MHC class I (Kanneganti, 2010; Meissner et al., 2010), mounted among pairwise comparisons (Table 2), speculating that this ISG may be a cross-talker in the synergetic antiviral effect induced by IFN-γ. Even more noteworthy, OASL possesses an antiviral effect against the single-stranded RNA virus (Marques et al., 2008) and can potentially be used to overcome viral evasion and enhance innate immunity (Zhu et al., 2015). It was upregulated in all pairwise comparisons (Table 2), suggesting that it may be a key molecule for IFN-γ to function in its anti-GETV activity. Previous studies have indicated that the mechanisms underlying the antiviral activity of IFN-γ may vary in different target cell types, and in ST cells, IRF1 plays a critical role in IFN-γ-induced inhibition against transmissible gastroenteritis virus (TGEV) (Shan et al., 2019). In contrast, *IRF1* was not significantly upregulated in our study. This inconsistent result may be caused by the different viruses used and requires further exploration and clarification.

Interestingly, a total of 124 DEGs shared in pairwise comparisons of GETV *vs*. mock and IFN-γ *vs*. mock presented the identical upregulated tendency (Figure 6A, Supplementary Figure S1), indicating that IFN-γ could strengthen the biological processes induced by GETV infection to play a role in controlling innate antiviral immunity, such as the PRR-related pathways (i.e., NLR and TLR signaling pathways) and its downstream pathways, including the TNF signaling pathway, viral protein interaction with cytokine and cytokine receptor, the IL-17 signaling pathway, and cytokine-cytokine receptor interaction (Figure 6C). Kajita reported that IFN-γ might contribute to the innate immune response to cutaneous viral infections by accelerating TLR3 expression and function in keratinocytes (Kajita et al., 2015). Although numerous DEGs involved in the TLR signaling pathway were identified, none of the TLRs themselves were identified as DEGs. This needs further investigation to be elucidated.

Compared to GETV infection, treatment with IFN-γ could activate additional immune-related genes, and the most significantly enriched pathways were antigen procession and presentation, cytokine-cytokine receptor interaction, complement and coagulation cascades, and the JAK-STAT signaling pathway (Figures 6D–F). It was, therefore, possible that the JAK-STAT signaling pathway, the classical pathway activated by interferons to initiate the transcription of ISGs, may also be indispensable to the antiviral effect of IFN-γ on alphavirus, which was supported by the mechanism for IFN-γ-mediated clearance of SINV infection from mature neurons (Platanias, 2005). Interestingly, several DEGs (i.e., *IFNE, IL22RA1*, and *OSM*) enriched in the cytokine-cytokine receptor interaction pathway were involved in the JAK-STAT signaling pathway in this study. Furthermore, complement and coagulation cascades play vital roles in initiating and regulating the innate immune system responses against virus infection, and the individual proteins of this pathway are strictly regulated (Maloney et al., 2020). In this study, DEGs involved in complement and coagulation cascades (i.e., *C2, SERPING1, C4A, C1R, C1S, C3, C5, CFH*, and *F2*) were upregulated in GETV-infected ST cells through treatment with IFN-γ (Figure 5B), indicating that the activation of the complement and coagulation cascades may be the pivotal mechanism for the IFN-γ-mediated inhibitory effect of GETV. To our knowledge, antigen processing and presentation, the cornerstones of adaptive immunity (Pishesha et al., 2022), are the traditional immunoregulatory functions of IFN-γ. However, they may solely play an inferior role in the process of defense against GETV replication by IFN-γ in non-immune ST cells. Therefore, it could be speculated that the activation of JAK-STAT signaling, coupled with complement and coagulation cascades, may play synergetic roles in the anti-GETV activity of IFN-γ in ST cells.

In summary, comparative transcriptome profiling between mock- and GETV-infected ST cells was obtained for the first time, and it was inferred that GETV could escape from some innate immune responses, which would help us reveal the precise mechanisms underlying virus-host interaction. Importantly, exogenous porcine IFN-γ showed a dose-dependent antiviral effect against GETV *in vitro*, which would contribute to the prevention and control of the virus. Furthermore, IFN-γ could exert biological processes analogous to those induced by GETV, and the primary mechanism for IFN-γ-mediated inhibition of GETV proliferation may be related to the synergetic effect of the JAK-STAT signaling pathway and complement and coagulation cascades; NLRC5 and OASL may be the cross-talkers during the synergetic antiviral effect induced by IFN-γ. The abovementioned mechanisms need to be addressed in the future, and the application prospects of IFN-γ on the prevention of GETV should be further investigated *in vivo*.

## Data availability statement

The datasets presented in this study can be found in online repositories. The names of the repository/repositories and accession number(s) can be found below: https://www.ncbi.nlm.nih.gov/, PRJNA799863.

## Author contributions

ZZ and YL designed and supervised the study. JL and XG wrote the preliminary draft manuscript. JL, XG, HS, TW, and WG performed experiments. XG, JL, XL, and HJ performed data analysis and created the figures and tables. ZZ, YL, XG, JL, and TW edited, revised, and finalized the manuscript. All authors contributed to the article and approved its submitted version.

## References

[B1] Bautista E. M. Molitor T. W. (1999). IFN gamma inhibits porcine reproductive and respiratory syndrome virus replication in macrophages. Arch. Virol. 144, 1191–1200. 10.1007/s007050050578 10446652

[B2] Burdeinick-Kerr R. Govindarajan D. Griffin D. E. (2009). Noncytolytic clearance of sindbis virus infection from neurons by gamma interferon is dependent on Jak/STAT signaling. J. Virol. 83, 3429–3435. 10.1128/JVI.02381-08 19176616 PMC2663278

[B3] Burdeinick-Kerr R. Griffin D. E. (2005). Gamma interferon-dependent, noncytolytic clearance of sindbis virus infection from neurons in vitro. J. Virol. 79, 5374–5385. 10.1128/JVI.79.9.5374-5385.2005 15827152 PMC1082728

[B4] Burke J. M. Moon S. L. Matheny T. Parker R. (2019). RNase L reprograms translation by widespread mRNA turnover escaped by antiviral mRNAs. Mol. Cell. 75, 1203–17. 10.1016/j.molcel.2019.07.029 31494035 PMC6754297

[B5] Chaudhary V. Yuen K. S. Chan J. F. Chan C. P. Wang P. H. Cai J. P.. (2017). Selective activation of type, I. interferon signaling by zika virus NS5 protein. J. Virol. 91, 14. 10.1128/JVI.00163-17 28468880 PMC5487581

[B6] Chen W. Foo S. S. Zaid A. Teng T. S. Herrero L. J. Wolf S.. (2017). Specific inhibition of NLRP3 in chikungunya disease reveals a role for inflammasomes in alphavirus-induced inflammation. Nat Microbiol. 2, 1435–1445. 10.1038/s41564-017-0015-4 28848230

[B7] Couderc T. Chretien F. Schilte C. Disson O. Brigitte M. Guivel-Benhassine F.. (2008). A mouse model for Chikungunya: young age and inefficient type-I interferon signaling are risk factors for severe disease. PLoS Pathog. 4, e29. 10.1371/journal.ppat.0040029 18282093 PMC2242832

[B8] Dhanwani R. Khan M. Bhaskar A. S. Singh R. Patro I. K. Rao P. V.. (2012). Characterization of Chikungunya virus infection in human neuroblastoma SH-SY5Y cells: role of apoptosis in neuronal cell death. Virus Res. 163, 563–572. 10.1016/j.virusres.2011.12.009 22210004

[B9] Fan W. Jiao P. Zhang H. Chen T. Zhou X. Qi Y.. (2020). Inhibition of African swine fever virus replication by porcine type I and type II interferons. Front. Microbiol. 11, 1203. 10.3389/fmicb.2020.01203 32655518 PMC7325991

[B10] Floyd-Smith G. Slattery E. Lengyel P. (1981). Interferon action: RNA cleavage pattern of a (2′-5′)oligoadenylate–dependent endonuclease. Science. 212, 1030–1032. 10.1126/science.6165080 6165080

[B11] Frolova E. I. Fayzulin R. Z. Cook S. H. Griffin D. E. Rice C. M. Frolov I.. (2002). Roles of nonstructural protein nsP2 and Alpha/Beta interferons in determining the outcome of Sindbis virus infection. J. Virol. 76, 11254–11264. 10.1128/JVI.76.22.11254-11264.2002 12388685 PMC136776

[B12] Fros J. J. Major L. D. Scholte F. E. M. Gardner J. van Hemert M. J. Suhrbier A.. (2015). Chikungunya virus non-structural protein 2-mediated host shut-off disables the unfolded protein response. J. Gen. Virol. 96, 580–589. 10.1099/vir.0.071845-0 25395592

[B13] Fros J. J. Pijlman G. P. (2016). Alphavirus infection: host cell shut-off and inhibition of antiviral responses. Viruses. 8, 166. 10.3390/v8060166 27294951 PMC4926186

[B14] Garcia-Sastre A. (2017). Ten strategies of interferon evasion by viruses. Cell Host Microbe. 22, 176–184. 10.1016/j.chom.2017.07.012 28799903 PMC5576560

[B15] Griffin D. E. Hardwick J. M. (1997). Regulators of apoptosis on the road to persistent alphavirus infection. Annu. Rev. Microbiol. 51, 565–592. 10.1146/annurev.micro.51.1.565 9343360

[B16] Hamana A. Takahashi Y. Uchida T. Nishikawa M. Imamura M. Chayama K.. (2017). Evaluation of antiviral effect of type I, II, and III interferons on direct-acting antiviral-resistant hepatitis C virus. Antiviral Res. 146, 130–138. 10.1016/j.antiviral.2017.08.017 28864074

[B17] Hwang S. Y. Hertzog P. J. Holland K. A. Sumarsono S. H. Tymms M. J. Hamilton J. A.. (1995). A null mutation in the gene encoding a type I interferon receptor component eliminates antiproliferative and antiviral responses to interferons alpha and beta and alters macrophage responses. Proc. Natl. Acad. Sci. USA. 92, 11284–11288. 10.1073/pnas.92.24.11284 7479980 PMC40616

[B18] Jiao H. Wachsmuth L. Kumari S. Schwarzer R. Lin J. Eren R. O.. (2020). Z-nucleic-acid sensing triggers ZBP1-dependent necroptosis and inflammation. Nature. 580, 391–395. 10.1038/s41586-020-2129-8 32296175 PMC7279955

[B19] Kajita A. I. Morizane S. Takiguchi T. Yamamoto T. Yamada M. Iwatsuki K.. (2015). Interferon-gamma enhances TLR3 expression and antiviral activity in keratinocytes. J. Invest. Dermatol. 135, 2005–2011. 10.1038/jid.2015.125 25822580

[B20] Kanneganti T. D. (2010). Central roles of NLRs and inflammasomes in viral infection. Nat. Rev. Immunol. 10, 688–698. 10.1038/nri2851 20847744 PMC3909537

[B21] Kato H. Sato S. Yoneyama M. Yamamoto M. Uematsu S. Matsui K.. (2005). Cell type-specific involvement of RIG-I in antiviral response. Immunity 23, 19–28. 10.1016/j.immuni.2005.04.010 16039576

[B22] Kato H. Takeuchi O. Sato S. Yoneyama M. Yamamoto M. Matsui K.. (2006). Differential roles of MDA5 and RIG-I helicases in the recognition of RNA viruses. Nature 441, 101–105. 10.1038/nature04734 16625202

[B23] Kawasaki T. Kawai T. (2014). Toll-like receptor signaling pathways. Front. Immunol. 5, 461. 10.3389/fimmu.2014.00461 25309543 PMC4174766

[B24] Li F. Zhang B. Xu Z. Jiang C. Nei M. Xu L.. (2022). Getah virus infection rapidly causes testicular damage and decreases sperm quality in male mice. Front. Vet. Sci. 9, 883607. 10.3389/fvets.2022.883607 35548045 PMC9083227

[B25] Li L. Fu F. Xue M. Chen W. Liu J. Shi H.. (2017). IFN-lambda preferably inhibits PEDV infection of porcine intestinal epithelial cells compared with IFN-alpha. Antiviral Res. 140, 76–82. 10.1016/j.antiviral.2017.01.012 28109912 PMC7113730

[B26] Li X. D. Qiu F. X. Yang H. Rao Y. N. Calisher C. H. (1992). Isolation of Getah virus from mosquitos collected on Hainan Island, China, and results of a serosurvey. Southeast Asian J. Trop. Med. Public Health. 23, 730–4. 1338481

[B27] Li Y. Fu S. Guo X. Li X. Li M. Wang L.. (2019). Serological survey of getah virus in domestic animals in Yunnan Province, China. Vector Borne Zoonotic Dis. 19, 59–61. 10.1089/vbz.2018.2273 29957135

[B28] Linder A. Bothe V. Linder N. Schwarzlmueller P. Dahlström F. Bartenhagen C.. (2021). Defective interfering genomes and the full-length viral genome trigger RIG-I after infection with vesicular stomatitis virus in a replication dependent manner. Front. Immunol. 12, 595390. 10.3389/fimmu.2021.595390 33995343 PMC8119886

[B29] Liu M. Jia S. Dong T. Zhao F. Xu T. Yang Q.. (2020). Metabolomic and transcriptomic analysis of MCF-7 cells exposed to 23 chemicals at human-relevant levels: estimation of individual chemical contribution to effects. Environ. Health Perspect. 128, 127008. 10.1289/EHP6641 33325755 PMC7741182

[B30] Liu X. Wei Y. Li Y. Li H. Yang X. Yi Y.. (2016). A highly efficient and simple construction strategy for producing recombinant baculovirus Bombyx mori nucleopolyhedrovirus. PLoS ONE. 11, e0152140. 10.1371/journal.pone.0152140 27008267 PMC4805210

[B31] Lu G. Chen R. Shao R. Dong N. Liu W. Li L. S.. (2020). Getah virus: an increasing threat in China. J. Infect. 80, 350–371. 10.1016/j.jinf.2019.11.016 31790706

[B32] Lu G. Ou J. Ji J. Ren Z. Hu X. Wang C.. (2019). Emergence of Getah virus infection in horse with fever in China, 2018. Front. Microbiol. 10, 1416. 10.3389/fmicb.2019.01416 31281304 PMC6596439

[B33] Maloney B. E. Perera K. D. Saunders D. R. D. Shadipeni N. Fleming S. D. (2020). Interactions of viruses and the humoral innate immune response. Clin. Immunol. 212, 108351. 10.1016/j.clim.2020.108351 32028020 PMC7062564

[B34] Marchette N. J. Rudnick A. Garcia R. (1980). Alphaviruses in peninsular Malaysia: II. serological evidence of human infection. Southeast Asian J. Trop. Med. Public Health 11, 14–23. https://pubmed.ncbi.nlm.nih.gov/?term=Rudnick+A&cauthor_id=7403943 7403943

[B35] Marques J. Anwar J. Eskildsen-Larsen S. Rebouillat D. Paludan S. R. Sen G.. (2008). The p59 oligoadenylate synthetase-like protein possesses antiviral activity that requires the C-terminal ubiquitin-like domain. J. General Virol. 89, 2767–2772. 10.1099/vir.0.2008/003558-0 18931074

[B36] Matsumoto M. Seya T. (2008). TLR3: interferon induction by double-stranded RNA including poly(I:C). Adv. Drug Deliv. Rev. 60, 805–812. 10.1016/j.addr.2007.11.005 18262679

[B37] Meissner T. B. Li A. Biswas A. Lee K. H. Liu Y. J. Bayir E.. (2010). NLR family member NLRC5 is a transcriptional regulator of MHC class I genes. Proc. Natl. Acad. Sci. USA. 107, 13794–13799. 10.1073/pnas.1008684107 20639463 PMC2922274

[B38] Nelemans T. Kikkert M. (2019). Viral Innate Immune Evasion and the Pathogenesis of Emerging RNA Virus Infections. Viruses *(2019)* 11(10). 10.3390/v11100961 31635238 PMC6832425

[B39] Nemoto M. Bannai H. Tsujimura K. Kobayashi M. Kikuchi T. Yamanaka T.. (2015). Getah virus infection among racehorses, Japan, 2014. Emerging Infect. Dis. 21, 883–885. 10.3201/eid2105.141975 25898181 PMC4412242

[B40] Nigg P. E. Pavlovic J. (2015). Oligomerization and GTP-binding requirements of MxA for viral target recognition and antiviral activity against influenza A virus. J. Biol. Chem. 290, 29893–29906. 10.1074/jbc.M115.681494 26507657 PMC4706002

[B41] Pichlmair A. Schulz O. Tan C. P. Rehwinkel J. Kato H. Takeuchi O.. (2009). Activation of MDA5 requires higher-order RNA structures generated during virus infection. J. Virol. 83, 10761–10769. 10.1128/JVI.00770-09 19656871 PMC2753146

[B42] Pishesha N. Harmand T. J. Ploegh H. L. (2022). A guide to antigen processing and presentation. Nat. Rev. Immunol. 22, 751–764. 10.1038/s41577-022-00707-2 35418563

[B43] Platanias L. C. (2005). Mechanisms of type-I- and type-II-interferon-mediated signalling. Nat. Rev. Immunol. 5, 375–386. 10.1038/nri1604 15864272

[B44] Ramana C. V. Gil M. P. Schreiber R. D. Stark G. R. (2002). Stat1-dependent and -independent pathways in IFN-γ-dependent signaling. Trends Immunol. 23, 96–101. 10.1016/S1471-4906(01)02118-4 11929133

[B45] Reed L. J. Muench H. A. (1938). Simple method of estimating fifty per cent endpoints12. Am. J. Epidemiol. 27, 493–497. 10.1093/oxfordjournals.aje.a118408

[B46] Rhein B. A. Powers L. S. Rogers K. Anantpadma M. Singh B. K. Sakurai Y.. (2015). Interferon-gamma inhibits ebola virus infection. PLoS Pathog. 11, e1005263. 10.1371/journal.ppat.1005263 26562011 PMC4643030

[B47] Ryman K. D. Klimstra W. B. Nguyen K. B. Biron C. A. Johnston R. E. (2000). Alpha/beta interferon protects adult mice from fatal Sindbis virus infection and is an important determinant of cell and tissue tropism. J. Virol. 74, 3366–3378. 10.1128/JVI.74.7.3366-3378.2000 10708454 PMC111838

[B48] Saito T. Owen D. M. Jiang F. Marcotrigiano J. Gale M. (2008). Jr. Innate immunity induced by composition-dependent RIG-I recognition of hepatitis C virus RNA. Nature. 454, 523–527. 10.1038/nature07106 18548002 PMC2856441

[B49] Schattgen S. A. Oguin T. H. Thomas P. G. (2016). The antiviral molecule Mx1 positively regulates the induction of type I IFN in response to influenza infection. J. Immunol. 196, 202–207. 10.4049/jimmunol.196.Supp.202.7

[B50] Schilte C. Couderc T. Chretien F. Sourisseau M. Gangneux N. Guivel-Benhassine F.. (2010). Type I IFN controls chikungunya virus via its action on nonhematopoietic cells. J. Exp. Med. 207, 429–442. 10.1084/jem.20090851 20123960 PMC2822618

[B51] Schoggins J. W. (2019). Interferon-stimulated genes: what do they all do? Annu. Rev. Virol. 6, 567–584. 10.1146/annurev-virology-092818-015756 31283436

[B52] Shan L. Fu F. Xue M. Zhu X. Li L. Feng L.. (2019). Interferon gamma inhibits transmissible gastroenteritis virus infection mediated by an IRF1 signaling pathway. Arch. Virol. 164, 2659–2669. 10.1007/s00705-019-04362-2 31385116 PMC7086799

[B53] Shi N. Li L. X. Lu R. G. Yan X. J. Liu H. (2019). Highly pathogenic swine Getah virus in blue foxes, Eastern China, 2017. Emerging Infect. Dis. 25, 1252–1254. 10.3201/eid2506.181983 31107236 PMC6537705

[B54] Takeuchi O. Akira S. (2009). Innate immunity to virus infection. Immunol. Rev. 227, 75–86. 10.1111/j.1600-065X.2008.00737.x 19120477 PMC5489343

[B55] Takeuchi O. Akira S. (2010). Pattern recognition receptors and inflammation. Cell. 140, 805–820. 10.1016/j.cell.2010.01.022 20303872

[B56] Thompson M. R. Kaminski J. J. Kurt-Jones E. A. Fitzgerald K. A. (2011). Pattern recognition receptors and the innate immune response to viral infection. Viruses. 3, 920–940. 10.3390/v3060920 21994762 PMC3186011

[B57] Varfolomeev E. Vucic D. (2018). Intracellular regulation of TNF activity in health and disease. Cytokine. 101, 26–32. 10.1016/j.cyto.2016.08.035 27623350

[B58] Verhelst J. Van Hoecke L. Spitaels J. De Vlieger D. Kolpe A. Saelens X.. (2017). Chemical-controlled activation of antiviral myxovirus resistance protein 1. J. Biol. Chem. 292, 2226–2236. 10.1074/jbc.M116.748806 28011636 PMC5313096

[B59] Wang H. Gort T. Boyle D. L. Clem R. J. (2012). Effects of manipulating apoptosis on Sindbis virus infection of Aedes aegypti mosquitoes. J. Virol. 86, 6546–6554. 10.1128/JVI.00125-12 22438551 PMC3393565

[B60] Wang N. Zhai X. Li X. Wang Y. He W. T. Jiang Z.. (2022). Attenuation of Getah virus by a single amino acid substitution at residue 253 of the E2 protein that might be part of a new heparan sulfate binding site on alphaviruses. J. Virol. 96, e0175121. 10.1128/jvi.01751-21 34986000 PMC8941864

[B61] Yang T. Li R. Hu Y. Yang L. Zhao D. Du L.. (2018). An outbreak of Getah virus infection among pigs in China, 2017. Transbound. Emerg. Dis. 65, 632–7. 10.1111/tbed.12867 29575687

[B62] Yoneyama M. Kikuchi M. Matsumoto K. Imaizumi T. Miyagishi M. Taira K.. (2005). Shared and unique functions of the DExD/H-box helicases RIG-I, MDA5, and LGP2 in antiviral innate immunity. J. Immunol. 175, 2851–2858. 10.4049/jimmunol.175.5.2851 16116171

[B63] Zhu J. Z. Ghosh A. Sarkar S. N. (2015). OASL - a new player in controlling antiviral innate immunity. Curr. Opin. Virol. 12, 15–19. 10.1016/j.coviro.2015.01.010 25676874 PMC4470762

