# Supplementary material for: The host transcriptome change involved in the inhibitory effect of exogenous interferon-γ on Getah virus replication
Source: Front Microbiol. 2023 Jun 28;14:1214281. doi: 10.3389/fmicb.2023.1214281 (PMC10337660; doi:10.3389/fmicb.2023.1214281)
Supplement: Supplementary file 1 [file Data_Sheet_1.ZIP › Supplementary material/Supplementary Figure Captions.docx]

**Supplementary Figure**

**Figure S1**

**
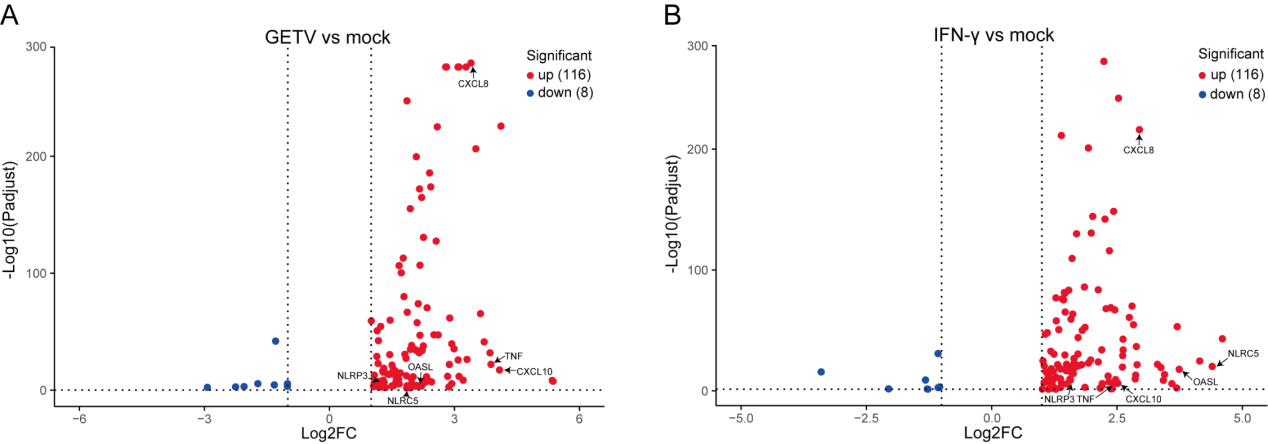
**

**Figure S1.** The common 124 DEGs between GETV *vs* mock and IFN-γ *vs* mock. **(A)**Volcano plots of the common 124 DEGs in GETV *vs* mock. (B) Volcano plots of the common 124 DEGs in IFN-γ *vs* mock. FC, fold change.

**Figure S2**

**
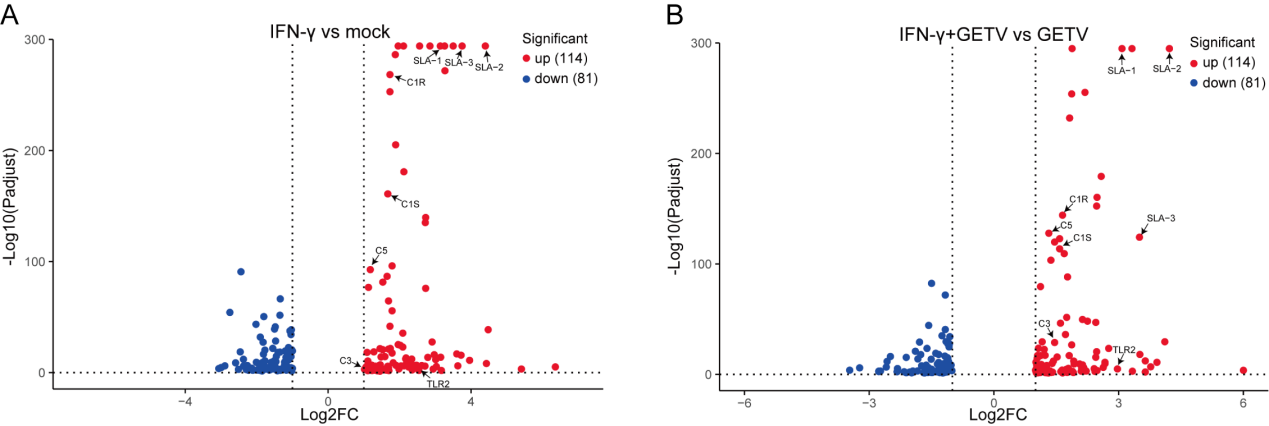
**

**Figure S2.** The common 195 DEGs between IFN-γ *vs* mock and IFN-γ+ GETV *vs* GETV. **(A)** Volcano plots of the common 195 DEGs in IFN-γ *vs* mock. **(B)** Volcano plots of the common 195 DEGs in IFN-γ+ GETV *vs* GETV. FC, fold change.

**Figure S3**

**
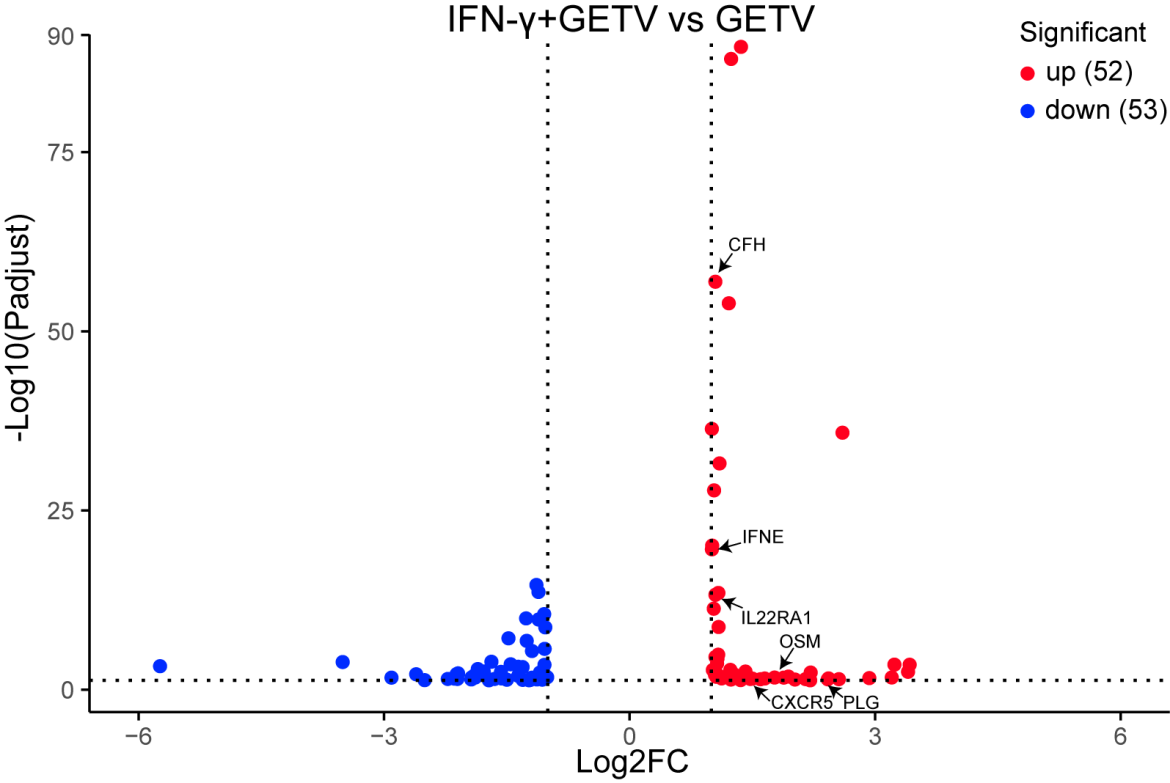
**

**Figure S3.** The 105 DEGs presented in IFN-γ+GETV *vs* GETV group but nor in other two groups. FC, fold change
