# Supplementary figures and images for: The host transcriptome change involved in the inhibitory effect of exogenous interferon-γ on Getah virus replication
Source: Front Microbiol. 2023 Jun 28;14:1214281. doi: 10.3389/fmicb.2023.1214281 (PMC10337660; doi:10.3389/fmicb.2023.1214281)

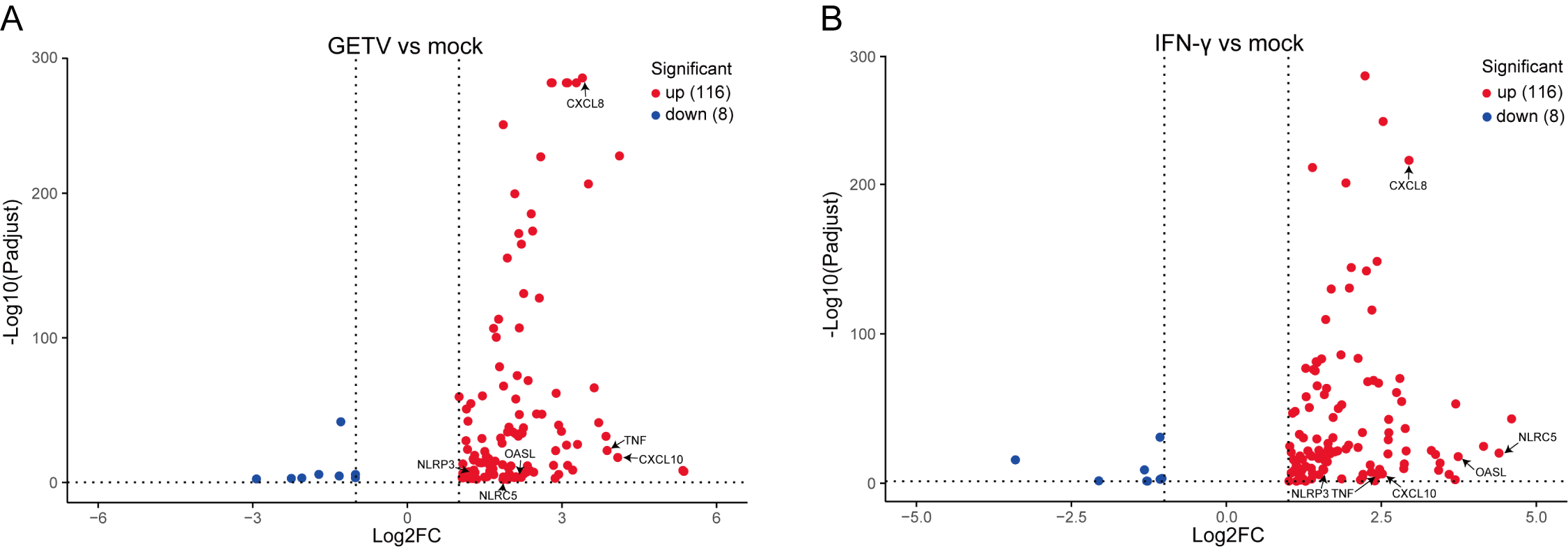

Supplement: Supplementary file 1 [file Data_Sheet_1.ZIP › Supplementary material/Figure S1.tif]

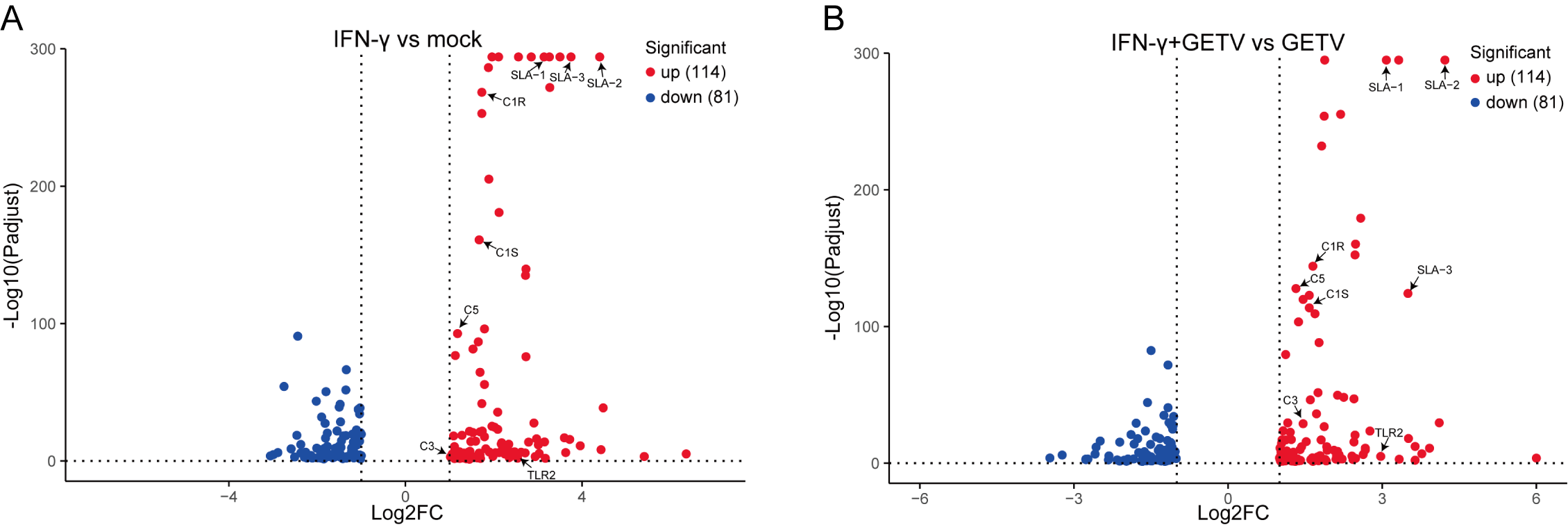

Supplement: Supplementary file 1 [file Data_Sheet_1.ZIP › Supplementary material/Figure S2.tif]

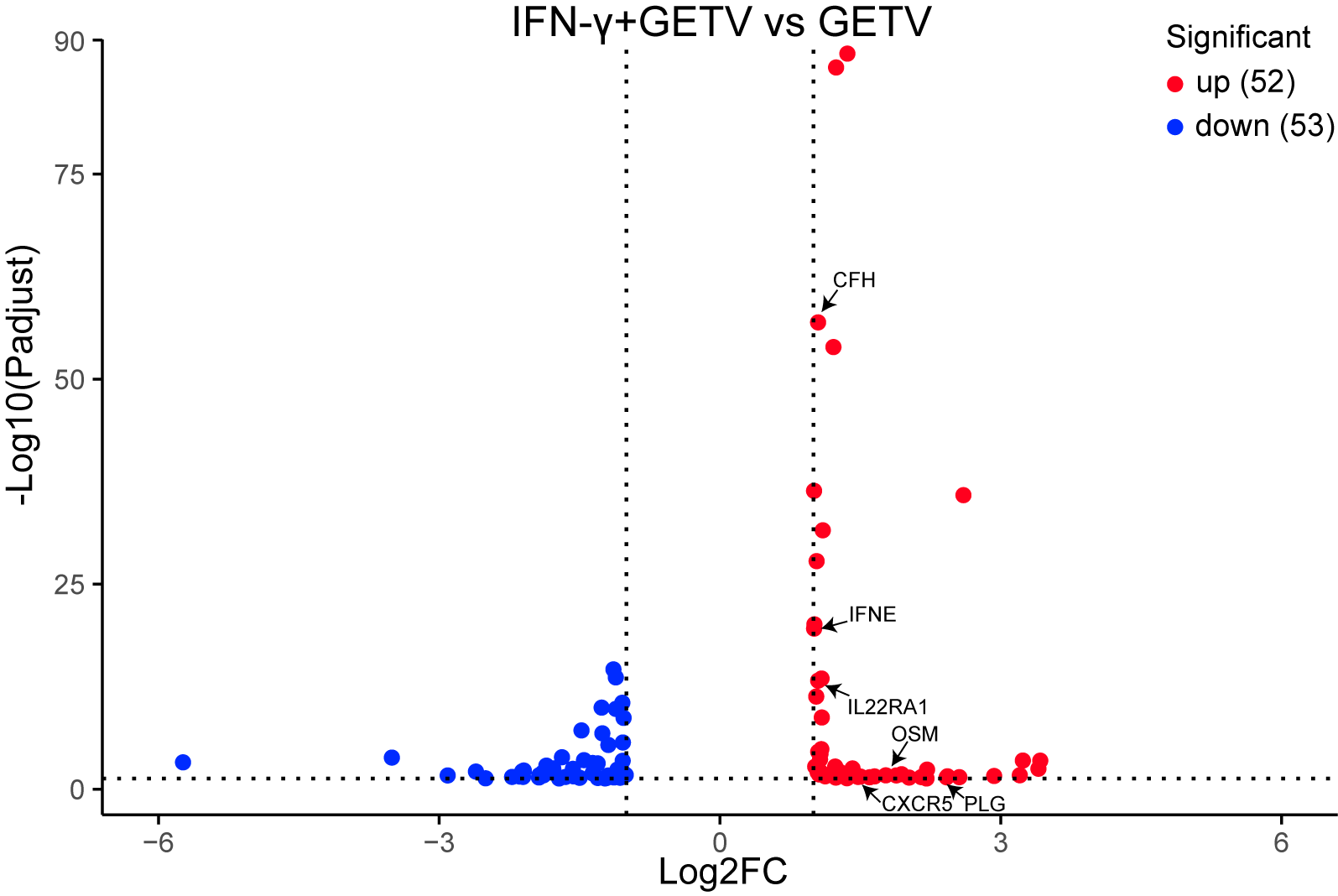

Supplement: Supplementary file 1 [file Data_Sheet_1.ZIP › Supplementary material/Figure S3.tif]
