# Supplementary material for: The host transcriptome change involved in the inhibitory effect of exogenous interferon-γ on Getah virus replication
Source: Front Microbiol. 2023 Jun 28;14:1214281. doi: 10.3389/fmicb.2023.1214281 (PMC10337660; doi:10.3389/fmicb.2023.1214281)
Supplement: Supplementary file 1 [file Data_Sheet_1.ZIP › Supplementary material/Supplementary Table.doc]

**Table S1：**

Primer sequences used in this study

| Gene name | Primer sequence (5'-3') | Application |
| --- | --- | --- |
| nsp3 | F: AGGGCGGTCGCTAAGAAG  R: TACGGTGGAGAAGTTTGGTC | RT-qPCR |
| CCL20 | F: AGCAACTTTGACTGCTGCCT  R: GATCTGCACACACGGCTAAC | RT-qPCR |
| PLAU | F: CATCCCGCTCCATACAGACC  R: CTGGATAGAGGTAGTCAGAGGGA | RT-qPCR |
| SULTIE1 | F: CCTGGTGGGAAAAGAGAACGAA  R: TCACCTCTTTTCTGATATTCTCCTT | RT-qPCR |
| TP53NP1 | F: CAGAAGACGAGGAGGAAGACA  R: TGGAGGAAGCAGGAATCACTT | RT-qPCR |
| POCIA | F: GGCCTCTGACGACAAGACAA  R: CCATCCGGGGAGAACTTGG | RT-qPCR |
| SLA2 | F: GCAGGACTATTGGGATCGGG  R: GGATGGTGTGAGACCCGGC | RT-qPCR |
| DDX58 | F: TTGCTCAGTGCAATCTGGTC  R: CTTCCTCTGCCTCTGGTTTG | RT-qPCR |
| DHX58 | F: CCACCAAGACCCAGATCCTA  R: GACCCTTGAACTGCTTCTGC | RT-qPCR |
| IFIH1 | F: CAGTGTGCTAGCCTGCTCTG  R: GCAGTGCCTTGTTTCCTCTC | RT-qPCR |
| ZBP1 | F: GAGTCCAGAGGCTTCCATTGA  R: GGCTGACGCTCATCCTGTT | RT-qPCR |
| TNF | F: CGACTCAGTGCCGAGATCAA  R: CTCACAGGGCAATGATCCCA | RT-qPCR |
| IL-1α | F: TGGCTGCTGTGCTAAATAAC  R: CAGGAAGTTGCGAATCATC | RT-qPCR |
| IL-6 | F: ATCAGGAGACCTGCTTGATG  R: TGGTGGCTTTGTCTGGATTC | RT-qPCR |
| CXCL10 | F: TGCCCACATGTTGAGATCAT  R: CGGCCCATCCTTATCAGTAG | RT-qPCR |
| TNFAIP3 | F: TTTGTCCCCCTGGTGACCCTGA  R: TTTCGGGATCTGTCAAGAAGTGAAC | RT-qPCR |
| ZBP1 | F: GAGTCCAGAGGCTTCCATTGA  R: GGCTGACGCTCATCCTGTT | RT-qPCR |
| NLRP3 | F: CAGCAGATGGATAGCGGCAAGAG  R: CAGTGAGCAGAGACCCCAGAGG | RT-qPCR |
| GADD45B | F: GGACTTAGACTTTGGGACTTG  R: GTAAGCCTCCCATCTCTCTT | RT-qPCR |
| PMAIP1 | F: GGGCGTTCAGGGTGTTGCTTT  R: CGAGACCTCCTTCCAGGCATCT | RT-qPCR |
| MCL1 | F: GCATCGAACCGTTAGCAGA  R: GAAGAACTCCACAAACCCATC | RT-qPCR |
| ZFP36 | F: CCTCCCGCTACAAGACTGAG  R: ACTTGTGGCAGAGTTCCGTC | RT-qPCR |
| IFIT3 | F: GCAGCCAAATTTTACCGAGT  R: AGTCCTTGGCATATTTCCGTA | RT-qPCR |
| MX1 | F: AGTGTCGGCTGTTTACCAAG  R: TTCACAAACCCTGGCAACTC | RT-qPCR |
| ISG15 | F: GGTGAGGAACGACAAGGGTC  R: GGCTTGAGGTCATACTCCCC | RT-qPCR |
| OAS1 | F: AACCCAACCCGGAAATCTAC  R: GCTCTTCAGCTTGGTTGGTC | RT-qPCR |
| IFN-λ3 | F: GCCCAGTTCAAGTCTCTGTC  R: GCAGTTCCAGTCCTCCAAGA | RT-qPCR |
| IFN-α | F: CTGCTGCCTGGAATGAGAGCC  R: TGACACAGGCTTCCAGGTCCC | RT-qPCR |
| IFN-ε | F: GTTGGTACTGCTGGCTTCTTC  R: ACACTGCTGAATTGATGAGGTC | RT-qPCR |
| IFN-ω | F: CTGCGTCAGATGAGGAGAATT  R: TGTGGAGGAGGAGGAAGGT | RT-qPCR |
| CXCL8 | F: AGCCCGTGTCAACATGACTT  R: TGGAAAGGTGTGGAATGCGT | RT-qPCR |
| SLA-DQB1 | F: CAGCGGCGAGTGCAACCTAC  R: GATGGGGTTCTGGAGGCTGG | RT-qPCR |
| PLCL1 | F: GCCAATTCAGCAGATGTAGCA  R: ACAGAGGTGTCTTCCAGCATAA | RT-qPCR |
| CDKN1C | F: ACGAGGAGCTGAGTCGTGAG  R: CTGTCCACCTCGGTCCACT | RT-qPCR |
| NCBP2 | F: GCCACCAGGGAACTCAGAT  R: GACCATCAGCTCACAATCAAGA | RT-qPCR |
| ZNF484 | F: TGTGGCAGCAGGTAACTCAT  R: AGTCTGTAACTCGTGGCTAAGT | RT-qPCR |
| SLA-1 | F: ACTCTCGCTTCATCGCCGTC  R: CCCACTCCGTAAGTCTGTGC | RT-qPCR |
| SLA-3 | F: GGAAGCCCCGTTTCACCTC  R: TCAGGTTCACTCGGAAAGTC | RT-qPCR |
| SLA-DRA | F: CCCGCCAGTGGTCAATGT  R: AGTGGAACTTGCGGAAAAGG | RT-qPCR |
| SLA-DRB1 | F: GAGGGCACGGTCTGAATC  R: AGGGCGTCCTTTCTGATT | RT-qPCR |
| SLA-DQA1 | F: GCCTACAAGAGCTGAAGAGTGGATGT  R: TGAGGGATCAGGTAGCCGAGTG | RT-qPCR |
| C1R | F: GCAGCCTCAGTACGAGTTCC  R: GACAGTAGCACCTGCTTCCC | RT-qPCR |
| C2 | F: GAGGGTCAGTGTGGGGGA  R: TCCCTGATGCTTCTTGGCAG | RT-qPCR |
| C5 | F: GTGACCTTCGACGTGCTTCCTC  R: CACCTTCTGGCACCACTCGTAATG | RT-qPCR |
| PLG | F: AATGCTGGCTTGACAATGAACT  R: ACTCTTGGATCGGTGGTGTAG | RT-qPCR |
| F2 | F: TCTGGAAGGTAACTGTGCTGAA  R: GGTGGAGTTGACTTCTGGCTTA | RT-qPCR |
| NLRC5 | F: AACTTGGCCGAGAACAGC  R: TTCCACAGGCGAATGACTT | RT-qPCR |
| ISG12(A) | F: AGATACTGGCGACAGGGAGT  R: AGGGCAGCCTTGAATGACAG | RT-qPCR |
| DDIT3 | F: CTCTGGCTTGGCTGACTGA  R: TTGGTCTTCCTCCTCTTCCTC | RT-qPCR |
| RBP4 | F: CCCCGAGGGACTCTTTCTGC  R: TCTTGAACTTGGCAGGGTCC | RT-qPCR |
| IFI6 | F: AAGGCGGTATCGCTCTTCTT  R: GAGCTGCTGTTGTCCTCAGA | RT-qPCR |
| GAPDH | F: TCAACGACCACTTCGTCAAGC  R: GGATGGAAACTGGAAGTCAGGAGA | RT-qPCR |

**Table S2:**

DEGs in NOD-like receptor signaling pathway (GETV *vs* mock)

| Gene name | Log2FC(GETV/mock) | *P*_adjust_ |
| --- | --- | --- |
| TNFAIP3 | 2.809 | 0 |
| LOC100155195 | 2.254 | 2.99152E-05 |
| CXCL8 | 3.279 | 0 |
| CXCL2 | 2.587 | 1.0075E-222 |
| OAS1 | 1.931 | 0.001922046 |
| CCL5 | 2.172 | 2.60281E-45 |
| NLRP3 | 1.201 | 0.000338346 |
| LOC102157763 | 2.559 | 2.4396E-125 |
| NFKBIA | 1.860 | 8.0659E-245 |
| IL6 | 1.304 | 1.62202E-17 |
| OAS2 | 1.259 | 1.258555055 |
| LOC100525396 | 2.403 | 2.1971E-183 |
| TNF | 3.875 | 9.04363E-21 |

**Table S3:**

DEGs in Toll-like receptors signaling pathway (GETV *vs* mock)

| Gene name | Log2FC(GETV/mock) | *P*_adjust_ |
| --- | --- | --- |
| CXCL10 | 4.080 | 4.58089E-16 |
| CXCL8 | 3.279 | 0 |
| CCL4 | 3.110 | 8.38033E-11 |
| CCL5 | 2.172 | 2.60281E-45 |
| CXCL11 | 5.345 | 5.344771566 |
| PIK3R1 | 1.285 | 1.43865E-63 |
| NFKBIA | 1.860 | 8.0659E-245 |
| IL6 | 1.304 | 1.62202E-17 |
| MAP3K8 | 1.076 | 9.01598E-12 |
| TNF | 3.875 | 9.04363E-21 |

**Table S4:**

DEGs in RIG-I-like receptors signaling pathway (GETV *vs* mock)

| Gene name | Log2FC(GETV/mock) | *P*_adjust_ |
| --- | --- | --- |
| DDX58 | 1.203 | 0.00103023 |
| CXCL10 | 4.080 | 4.5809E-16 |
| CXCL8 | 3.279 | 0 |
| IFIH1 | 1.334 | 2.3449E-12 |
| NFKBIA | 1.860 | 8.066E-245 |
| DHX58 | 1.616 | 6.3869E-18 |
| ISG15 | 2.329 | 1.6229E-10 |
| TNF | 3.875 | 9.0436E-21 |

**Table S5:**

DEGs in NF-kappa B signaling pathway (GETV *vs* mock)

| Gene name | Log2FC(GETV/mock) | *P*_adjust_ |
| --- | --- | --- |
| DDX58 | 1.203 | 0.001030232 |
| TNFAIP3 | 2.809 | 0 |
| CXCL8 | 3.279 | 0 |
| CXCL2 | 2.587 | 1.0075E-222 |
| CCL4 | 3.110 | 8.38033E-11 |
| GADD45B | 2.097 | 6.60E-71 |
| GADD45G | 1.009 | 9.19E-23 |
| LOC102157763 | 2.559 | 2.44E-125 |
| NFKBIA | 1.860 | 8.07E-245 |
| ICAM1 | 1.038 | 1.56E-31 |
| PLAU | 3.397 | 4.67E-277 |
| VCAM1 | 1.600 | 1.12E-10 |
| LOC100525396 | 2.403 | 2.20E-183 |
| TNF | 3.875 | 9.04E-21 |

**Table S6:**

DEGs in TNF signaling pathway (GETV *vs* mock)

| Gene name | Log2FC(GETV/mock) | *P*_adjust_ |
| --- | --- | --- |
| TNFAIP3 | 2.809 | 0 |
| CCL20 | 2.876 | 1.06E-20 |
| LIF | 1.724 | 2.26E-98 |
| SOCS3 | 1.670 | 6.94E-05 |
| CXCL10 | 4.080 | 4.58E-16 |
| CXCL2 | 2.587 | 1.01E-222 |
| JUNB | 1.770 | 9.00E-111 |
| CCL5 | 2.172 | 2.60E-45 |
| LOC102157763 | 2.559 | 2.44E-125 |
| PIK3R1 | 1.285 | 1.44E-63 |
| NFKBIA | 1.860 | 8.07E-245 |
| IRF1 | 1.281 | 2.30E-41 |
| IL6 | 1.304 | 1.62E-17 |
| ICAM1 | 1.038 | 1.56E-31 |
| MAP3K8 | 1.076 | 9.02E-12 |
| VCAM1 | 1.600 | 1.12E-10 |
| LOC100525396 | 2.403 | 2.20E-183 |
| TNF | 3.875 | 9.04E-21 |

**Table S7:**

DEGs in IL-17 signaling pathway (GETV *vs* mock)

| Gene name | Log2FC(GETV/mock) | *P*_adjust_ |
| --- | --- | --- |
| TNFAIP3 | 2.809 | 0 |
| CCL20 | 2.876 | 1.06E-20 |
| CXCL10 | 4.080 | 4.58089E-16 |
| CXCL8 | 3.279 | 0 |
| CXCL2 | 2.587 | 1.0075E-222 |
| LOC102157763 | 2.559 | 2.4396E-125 |
| NFKBIA | 1.860 | 8.0659E-245 |
| IL6 | 1.304 | 1.62202E-17 |
| FOSL1 | 4.115 | 2.8421E-223 |
| FOSB | 1.304 | 3.57889E-15 |
| LOC100525396 | 2.403 | 2.1971E-183 |
| TNF | 3.875 | 9.04363E-21 |

**Table S8:**

DEGs in viral protein interaction with cytokine and cytokine receptor (GETV *vs* mock)

| Gene name | Log2FC(GETV/mock) | *P*_adjust_ |
| --- | --- | --- |
| CCL20 | 2.876 | 1.06E-20 |
| CXCL10 | 4.080 | 4.58089E-16 |
| CXCL8 | 3.279 | 0 |
| CXCL2 | 2.587 | 1.0075E-222 |
| CCL4 | 3.110 | 8.38033E-11 |
| CCL5 | 2.172 | 2.60281E-45 |
| LOC102157763 | 2.559 | 2.4396E-125 |
| CXCL11 | 5.345 | 5.344771566 |
| IL6 | 1.304 | 1.62202E-17 |
| IL2RA | 1.312 | 3.33E-06 |
| LOC100525396 | 2.403 | 2.1971E-183 |
| TNF | 3.875 | 9.04363E-21 |

**Table S9:**

DEGs in cytokine-cytokine receptor interaction (GETV *vs* mock)

| Gene name | Log2FC(GETV/mock) | *P*_adjust_ |
| --- | --- | --- |
| CCL20 | 2.876 | 1.06E-20 |
| LIF | 1.724 | 2.26E-98 |
| TNFRSF12A | 1.079 | 6.89E-35 |
| CXCL10 | 4.080 | 4.5809E-16 |
| CXCL8 | 3.279 | 0 |
| CXCL2 | 2.587 | 1.008E-222 |
| CCL4 | 3.110 | 8.3803E-11 |
| GDF15 | 1.181 | 1.87E-34 |
| CCL5 | 2.172 | 2.6028E-45 |
| LOC102157763 | 2.559 | 2.44E-125 |
| IL11 | 3.301 | 6.11E-25 |
| CXCL11 | 5.345 | 5.34477157 |
| IL28B | 7.241 | 2.57E-06 |
| IL1A | 2.103 | 5.81E-56 |
| IL6 | 1.304 | 1.622E-17 |
| TNFSF15 | 1.702 | 1.36E-13 |
| IL2RA | 1.312 | 3.33E-06 |
| LOC100525396 | 2.403 | 2.197E-183 |
| TNF | 3.875 | 9.0436E-21 |

**Table S10:**

DEGs in chemokine signaling pathway (GETV *vs* mock)

| Gene name | Log2FC(GETV/mock) | *P*_adjust_ |
| --- | --- | --- |
| CCL20 | 2.876 | 1.06E-20 |
| CXCL10 | 4.080 | 4.58089E-16 |
| CXCL8 | 3.279 | 0 |
| CXCL2 | 2.587 | 1.0075E-222 |
| CCL4 | 3.110 | 8.38033E-11 |
| CCL5 | 2.172 | 2.60281E-45 |
| LOC102157763 | 2.559 | 2.44E-125 |
| CXCL11 | 5.345 | 5.344771566 |
| PIK3R1 | 1.285 | 1.44E-63 |
| NFKBIA | 1.860 | 8.07E-245 |
| LOC100525396 | 2.403 | 2.20E-183 |

**Table S11:**

DEGs in JAK-STAT signaling pathway (GETV *vs* mock)

| Gene name | Log2FC(GETV/mock) | *P*_adjust_ |
| --- | --- | --- |
| LIF | 1.724 | 2.26E-98 |
| MYC | 1.456 | 4.38E-58 |
| SOCS3 | 1.676 | 6.94E-05 |
| IL11 | 3.301 | 6.11E-25 |
| PIK3R1 | 1.285 | 1.44E-63 |
| IL28B | 7.241 | 2.57E-06 |
| IL6 | 1.304 | 1.62202E-17 |
| MCL1 | 1.119 | 4.08E-83 |
| IL2RA | 1.312 | 3.33E-06 |
| CISH | 1.421 | 1.420902963 |

**Table S12:**

DEGs in MAPK signaling pathway (GETV *vs* mock)

| Gene name | Log2FC(GETV/mock) | *P*_adjust_ |
| --- | --- | --- |
| AREG | 1.903 | 7.70174E-13 |
| EFNA1 | 1.053 | 1.10639E-16 |
| DUSP1 | 1.004 | 1.75113E-57 |
| MYC | 1.456 | 4.37507E-58 |
| DUSP10 | 2.152 | 1.51608E-30 |
| GADD45B | 2.097 | 6.60029E-71 |
| GADD45G | 1.009 | 9.18741E-23 |
| DUSP2 | 3.713 | 9.7594E-40 |
| IL1A | 2.103 | 5.80526E-56 |
| MAP3K8 | 1.076 | 9.01598E-12 |
| EPHA2 | 1.055 | 4.38105E-44 |
| DUSP4 | 1.263 | 0.014426959 |
| DUSP5 | 2.790 | 0 |
| EREG | 1.204 | 5.25703E-66 |
| TNF | 3.875 | 9.04363E-21 |

**Table S13:**

DEGs in cellular senescence (GETV *vs* mock)

| Gene name | Log2FC(GETV/mock) | *P*_adjust_ |
| --- | --- | --- |
| MYC | 1.456 | 4.38E-58 |
| ZFP36L2 | 1.120 | 7.44E-71 |
| CXCL8 | 3.279 | 0 |
| GADD45B | 2.097 | 6.60029E-71 |
| ETS2 | 1.531 | 1.85E-53 |
| GADD45G | 1.009 | 9.18741E-23 |
| PIK3R1 | 1.285 | 1.44E-63 |
| IL1A | 2.103 | 5.80526E-56 |
| IL6 | 1.304 | 1.62202E-17 |

**Table S14:**

DEGs in antigen processing and presentation (IFN-γ+GETV *vs* GETV)

| Gene name | Log2FC(IFN-γ+GETV/GETV) | *P*_adjust_ |
| --- | --- | --- |
| SLA-DMA | 1.074 | 2.00E-24 |
| B2M | 1.869 | 1.20E-254 |
| SLA-3 | 3.498 | 6.49E-125 |
| SLA-2 | 4.217 | 0 |
| SLA-1 | 3.085 | 0 |
| SLA-DQB1 | 1.692 | 4.44E-110 |
| LOC100515902 | 4.109 | 2.66E-30 |
| SLA-DRA | 2.187 | 4.83E-256 |
| CD74 | 3.079 | 0 |
| LOC100513601 | 3.320 | 0 |
| SLA-DRB1 | 1.366 | 4.02E-104 |
| SLA-DQA1 | 1.580 | 1.41E-123 |
| SLA-DMB | 1.208 | 2.15E-23 |
| HSPA6 | -1.352 | 0.004364597 |
| LOC102162186 | 1.889 | 0.001658229 |
| B2M | 1.878 | 1.12E-295 |
| SLA-DOA | 2.135 | 1.80E-50 |

**Table S15:**

DEGs in complement and coagulation cascades (IFN-γ+GETV *vs* GETV)

| Gene name | Log2FC(IFN-γ+GETV/GETV) | *P*_adjust_ |
| --- | --- | --- |
| CFH | 1.048 | 1.20E-57 |
| C4BPA | 2.065 | 3.35E-10 |
| SERPING1 | 1.819 | 8.14E-233 |
| C2 | 3.623 | 8.72E-183 |
| PLG | 2.430 | 0.026913173 |
| C3 | 1.462 | 1.26E-29 |
| F5 | -1.030 | 3.22E-09 |
| C5 | 1.321 | 1.47E-128 |
| F2 | 1.015 | 7.00E-06 |
| C4A | 1.817 | 1.817498335 |
| C1R | 1.648 | 7.15E-145 |
| C1S | 1.583 | 2.09E-114 |

**Table S16:**

DEGs in cell adhesion molecules (IFN-γ+GETV *vs* GETV)

| Gene name | Log2FC(IFN-γ+GETV/GETV) | *P*_adjust_ |
| --- | --- | --- |
| CD274 | 1.517 | 6.89E-08 |
| SLA-DMA | 1.074 | 2.00E-24 |
| SLA-3 | 3.498 | 6.49E-125 |
| SLA-2 | 4.217 | 0 |
| SLA-1 | 3.085 | 0 |
| SLA-DQB1 | 1.692 | 4.44E-110 |
| CDH3 | -1.139 | 2.46E-15 |
| LOC100515902 | 4.109 | 2.66E-30 |
| SLA-DRA | 2.187 | 4.83E-256 |
| LOC100513601 | 3.320 | 0 |
| SIGLEC1 | 2.557 | 0.034038542 |
| SLA-DRB1 | 1.366 | 4.02E-104 |
| SLA-DQA1 | 1.580 | 1.41E-123 |
| VSIR | 1.354 | 0.000704863 |
| SLA-DMB | 1.208 | 2.15E-23 |
| LOC102162186 | 1.889 | 0.001658229 |
| SLA-DOA | 2.135 | 1.80E-50 |

**Table S17:**

DEGs in cytokine-cytokine receptor interaction (IFN-γ+GETV *vs* GETV)

| Gene name | Log2FC(IFN-γ+GETV/GETV) | *P*_adjust_ |
| --- | --- | --- |
| GHR | -1.125 | 9.56E-05 |
| CCL20 | 1.557 | 1.78E-16 |
| IFNE | 1.005 | 2.63E-20 |
| CXCL10 | -1.723 | 0.02486647 |
| CXCL8 | 1.065 | 6.58E-37 |
| TNFSF13B | 1.933 | 3.20E-10 |
| CSF3 | 3.511 | 8.09E-19 |
| GDF1 | -1.133 | 0.0371585 |
| IL1R1 | 1.050 | 7.74E-18 |
| IL12RB1 | 1.285 | 0.00996674 |
| IL22RA1 | 1.049 | 5.80E-14 |
| CXCR2 | 2.180 | 0.00085442 |
| IL28B | -3.227 | 0.00068217 |
| IL1A | 1.576 | 7.21E-126 |
| CXCR5 | 1.502 | 0.0290638 |
| PPBP | 5.999 | 0.00018815 |
| IL15RA | 1.165 | 1.72E-08 |
| TNFSF14 | 1.280 | 0.03349483 |
| OSM | 1.771 | 0.02039997 |
| IL2RA | 1.293 | 6.14E-15 |
| AMCF-II | 1.748 | 2.36E-52 |

**Table S18:**

DEGs in phagosome (IFN-γ+GETV *vs* GETV)

| Gene name | Log2FC(IFN-γ+GETV/GETV) | *P*_adjust_ |
| --- | --- | --- |
| TLR2 | 2.973 | 1.08E-05 |
| C3 | 1.462 | 1.26E-29 |
| SLA-DMA | 1.074 | 2.00E-24 |
| SLA-3 | 3.498 | 6.49E-125 |
| SLA-2 | 4.217 | 0 |
| SLA-1 | 3.085 | 0 |
| SLA-DQB1 | 1.692 | 4.44E-110 |
| LOC100515902 | 4.109 | 2.66E-30 |
| SLA-DRA | 2.187 | 4.83E-256 |
| LOC100513601 | 3.320 | 0 |
| SLA-DRB1 | 1.366 | 4.02E-104 |
| SLA-DQA1 | 1.580 | 1.41E-123 |
| C1R | 1.648 | 7.15E-145 |
| NCF2 | 1.907 | 0.018751242 |
| SLA-DMB | 1.208 | 2.15E-23 |
| SLA-DOA | 2.135 | 1.80E-50 |

**Table S19:**

DEGs in antigen processing and presentation (IFN-γ *vs* mock)

| Gene name | Log2FC(IFN-γ/mock) | *P*_adjust_ |
| --- | --- | --- |
| SLA-DOB | 1.201 | 1.200693222 |
| TAP1 | 1.216 | 3.31E-69 |
| SLA-DMA | 1.534 | 3.11E-82 |
| B2M | 1.955 | 0 |
| SLA-3 | 3.752 | 0 |
| CTSS | 1.246 | 2.62E-15 |
| SLA-2 | 4.403 | 0 |
| SLA-1 | 3.138 | 0 |
| SLA-DQB1 | 2.115 | 1.34E-181 |
| LOC100515902 | 4.484 | 2.01E-39 |
| SLA-DRA | 2.561 | 0 |
| CD74 | 3.504 | 0 |
| LOC100513601 | 3.258 | 0 |
| SLA-DRB1 | 1.893 | 7.38E-206 |
| SLA-DQA1 | 2.111 | 8.11E-295 |
| CIITA | 1.481 | 6.94E-36 |
| SLA-DMB | 1.693 | 2.56E-65 |
| HSPA6 | -1.621 | 0.002116907 |
| LOC102162186 | 1.423 | 0.015105831 |
| B2M | 1.885 | 4.72E-287 |
| TNF | 2.437 | 2.93E-06 |
| SLA-DOA | 2.729 | 1.18E-76 |

**Table S20:**

DEGs in complement and coagulation cascades (IFN-γ *vs* mock)

| Gene name | Log2FC(IFN-γ/mock) | *P*_adjust_ |
| --- | --- | --- |
| LOC110259888 | 1.214 | 0.011224381 |
| C4BPA | 1.820 | 1.25E-09 |
| SERPING1 | 1.727 | 1.22E-253 |
| C2 | 4.600 | 8.28E-44 |
| CFB | 1.034 | 2.30E-33 |
| C3 | 1.062 | 6.93E-06 |
| F5 | -1.483 | 5.39E-42 |
| C5 | 1.177 | 1.72E-93 |
| F2 | 1.224 | 1.62E-07 |
| C4A | 2.620 | 1.44E-34 |
| C1R | 1.731 | 4.44E-269 |
| PLAU | 2.347 | 1.26E-116 |
| SERPINE1 | 1.928 | 1.23E-201 |
| C1S | 1.672 | 1.08E-161 |
| F3 | 1.972 | 2.95E-26 |

**Table S21:**

DEGs in cell adhesion molecules (IFN-γ *vs* mock)

| Gene name | Log2FC(IFN-γ/mock) | *P*_adjust_ |
| --- | --- | --- |
| SLA-DOB | 1.201 | 1.200693222 |
| ITGA8 | 1.092 | 1.67E-02 |
| CD274 | 2.606 | 2.61E+00 |
| SLA-DMA | 1.534 | 3.11E-82 |
| SLA-3 | 3.752 | 0 |
| SLA-2 | 4.403 | 0 |
| SLA-1 | 3.138 | 0 |
| SLA-DQB1 | 2.115 | 1.34E-181 |
| LOC100515902 | 4.484 | 2.01E-39 |
| SLA-DRA | 2.561 | 0 |
| LOC100513601 | 3.258 | 0 |
| SLA-DRB1 | 1.893 | 7.38E-206 |
| SLA-DQA1 | 2.111 | 8.11E-295 |
| CD80 | 3.576 | 0.005431703 |
| VCAM1 | 1.569 | 1.15E-09 |
| VSIR | 1.318 | 0.000216173 |
| SLA-DMB | 1.693 | 2.55548E-65 |
| LOC102162186 | 1.423 | 0.015105831 |
| SLA-DOA | 2.729 | 1.18E-76 |

**Table S22:**

DEGs in cytokine-cytokine receptor interaction (IFN-γ *vs* mock)

| Gene name | Log2FC(IFN-γ/mock) | *P*_adjust_ |
| --- | --- | --- |
| GHR | -1.223 | 1.18E-07 |
| CCL20 | 2.899 | 2.89892635 |
| LIF | 1.287 | 1.28651593 |
| CXCL10 | 2.515 | 2.51545099 |
| CXCL8 | 2.945 | 2.94541405 |
| TNFSF13B | 2.166 | 3.62E-13 |
| CSF3 | 2.622 | 3.90E-07 |
| CXCL2 | 2.430 | 3.56E-149 |
| CCL4 | 1.859 | 0.00111451 |
| IL1R1 | 0.001 | 1.62E-42 |
| CCL5 | 1.860 | 3.06E-53 |
| IL12RB1 | 1.504 | 0.00025132 |
| LOC102157763 | 2.374 | 1.63E-69 |
| IL11 | 1.374 | 0.00142294 |
| CXCL11 | 3.593 | 1.14E-06 |
| CXCR2 | 5.412 | 0.00046505 |
| IL1A | 2.885 | 2.35E-37 |
| IL6 | 1.458 | 2.11E-30 |
| PPBP | 2.938 | 0.00053948 |
| IL15RA | 1.116 | 1.10E-08 |
| TNFSF15 | 1.794 | 2.11E-22 |
| TNFSF14 | 3.012 | 1.58E-12 |
| IL2RA | 2.123 | 1.18E-24 |
| LOC100525396 | 2.259 | 8.84E-143 |
| AMCF-II | 2.725 | 7.86E-136 |
| TNF | 2.437 | 2.93E-06 |

**Table S23:**

DEGs in phagosome (IFN-γ *vs* mock)

| Gene name | Log2FC(IFN-γ/mock) | *P*_adjust_ |
| --- | --- | --- |
| SLA-DOB | 1.201 | 1.200693222 |
| TLR2 | 2.5108 | 2.510803795 |
| C3 | 1.062 | 6.93E-06 |
| TAP1 | 1.216 | 3.31E-69 |
| SLA-DMA | 1.534 | 3.11E-82 |
| SLA-3 | 3.752 | 0 |
| CTSS | 1.246 | 2.62E-15 |
| SLA-2 | 4.403 | 0 |
| SLA-1 | 3.138 | 0 |
| SLA-DQB1 | 2.115 | 1.34E-181 |
| LOC100515902 | 4.484 | 2.01E-39 |
| RAB7B | 1.48046 | 1.480459956 |
| SLA-DRA | 2.561 | 0 |
| LOC100513601 | 3.258 | 0 |
| SLA-DRB1 | 1.893 | 7.38E-206 |
| SLA-DQA1 | 2.111 | 8.11E-295 |
| C1R | 1.731 | 4.44E-269 |
| NCF2 | 1.45163 | 0.047412567 |
| SLA-DMB | 1.693 | 2.56E-65 |
| SLA-DOA | 2.729 | 1.18E-76 |

**Table S24:**

DEGs in IL-17 signaling pathway (IFN-γ *vs* mock)

| Gene name | Log2FC(IFN-γ/mock) | *P*_adjust_ |
| --- | --- | --- |
| TNFAIP3 | 1.691 | 1.10E-130 |
| CCL20 | 2.899 | 2.898926348 |
| MUC5AC | 1.785 | 7.21E-97 |
| MUC5B | 1.106 | 3.07E-11 |
| CXCL10 | 2.515 | 2.515450992 |
| CXCL8 | 2.945 | 2.945414047 |
| CSF3 | 2.622 | 3.90E-07 |
| FOS | 1.114 | 3.72E-86 |
| CXCL2 | 2.430 | 3.56E-149 |
| LCN2 | 1.154 | 0.007669355 |
| S100A8 | 1.696 | 0.013786182 |
| S100A9 | 1.628 | 0.005343858 |
| LOC102157763 | 2.374 | 1.63E-69 |
| NFKBIA | 1.460 | 1.80E-81 |
| IL6 | 1.458 | 2.11E-30 |
| FOSL1 | 1.674 | 1.85E-20 |
| FOSB | 1.378 | 9.08E-19 |
| LOC100525396 | 2.259 | 8.84E-143 |
| AMCF-II | 2.725 | 7.86E-136 |
| TNF | 2.437 | 2.93E-06 |

**Table S25:**

DEGs in TNF signaling pathway (IFN-γ *vs* mock)

| Gene name | Log2FC(IFN-γ/mock) | *P*_adjust_ |
| --- | --- | --- |
| TNFAIP3 | 1.691 | 1.10E-130 |
| CCL20 | 2.899 | 2.898926348 |
| LIF | 1.287 | 1.26E-58 |
| SOCS3 | 1.081 | 1.45E-16 |
| CXCL10 | 2.515 | 2.515450992 |
| FOS | 1.114 | 3.72E-86 |
| CXCL2 | 2.430 | 3.56E-149 |
| MAP2K6 | -1.847 | 7.79E-07 |
| JUNB | 1.448 | 4.49E-82 |
| CCL5 | 1.860 | 3.06E-53 |
| LOC102157763 | 2.374 | 1.63E-69 |
| NFKBIA | 1.460 | 1.80E-81 |
| IL6 | 1.458 | 2.11E-30 |
| MAP3K8 | 1.187 | 5.16E-19 |
| VCAM1 | 1.569 | 1.569379272 |
| LOC100525396 | 2.259 | 8.84E-143 |
| AMCF-II | 2.725 | 7.86E-136 |
| TNF | 2.437 | 2.93E-06 |

**Table S26:**

DEGs in Toll-like receptors signaling pathway (IFN-γ *vs* mock)

| Gene name | Log2FC(IFN-γ/mock) | *P*_adjust_ |
| --- | --- | --- |
| LBP | 1.070 | 3.75E-07 |
| TLR2 | 2.5108 | 2.510803795 |
| CXCL10 | 2.515 | 2.515450992 |
| CXCL8 | 2.945 | 2.945414047 |
| FOS | 1.114 | 3.72E-86 |
| CCL4 | 1.859 | 0.00111451 |
| LY96 | 1.103 | 7.77E-06 |
| SPP1 | 1.299 | 3.38E-145 |
| MAP2K6 | -1.847 | 7.79E-07 |
| CCL5 | 1.860 | 3.06E-53 |
| CXCL11 | 3.593 | 1.14E-06 |
| NFKBIA | 1.460 | 1.80E-81 |
| IL6 | 1.458 | 2.11E-30 |
| CD80 | 3.576 | 0.005431703 |
| MAP3K8 | 1.187 | 5.16E-19 |
| TNF | 2.437 | 2.93E-06 |

**Table S27:**

DEGs in Hematopoietic cell lineage (IFN-γ+GETV vs GETV)

| Gene name | Log2FC(FN-γ+GETV/GETV) | *P*_adjust_ |
| --- | --- | --- |
| SLA-DMA | 1.074 | 2.00E-24 |
| CSF3 | 3.511 | 8.09E-19 |
| IL1R1 | 1.050 | 7.73777E-18 |
| CD38 | 2.668 | 1.84E-10 |
| SLA-DQB1 | 1.692 | 4.4409E-110 |
| SLA-DRA | 2.187 | 4.83E-256 |
| IL1A | 1.576 | 7.21E-126 |
| SLA-DRB1 | 1.366 | 4.02E-104 |
| SLA-DQA1 | 1.580 | 1.41E-123 |
| ITGA2B | -1.307 | 7.05E-04 |
| SLA-DMB | 1.208 | 2.15E-23 |
| IL2RA | 1.293 | 6.14E-15 |
| SLA-DOA | 2.135 | 1.80E-50 |

**Table S28:**

The GO analyses of the 53 downregulated DEGs presented in IFN-γ+GETV *vs* GETV group but nor in other two groups obtained from the results of the Venn diagram analysis

| Biological process | Gene number | *P*_adjust_ |
| --- | --- | --- |
| Semaphorin-plexin signaling pathway involved in axon guidance | 2 | 0.1357 |
| Semaphorin-plexin signaling pathway involved in neuron projection guidance | 2 | 0.1357 |
| Negative regulation of developmental process | 8 | 0.1357 |
| Cell surface receptor signaling pathway | 11 | 0.1357 |
| Regulation of transforming growth factor beta2 production | 2 | 0.1593 |
